# Supplementary material for: Did an urban perinatal health programme in Rotterdam, the Netherlands, reduce adverse perinatal outcomes? Register-based retrospective cohort study
Source: BMJ Open. 2019 Oct 22;9(10):e031357. doi: 10.1136/bmjopen-2019-031357 (PMC6830581; doi:10.1136/bmjopen-2019-031357)
Supplement: Supplementary data [file bmjopen-2019-031357supp004.pdf]

Supplementary file 4. Logistic regression models that test the parallel trend assumption for perinatal mortality, small for gestational age and preterm birth during the preintervention period 2003-2009. Perinatal mortality is defined as still birth from 24 weeks onwards plus early neonatal mortality. Preterm is defined as born before a gestational age of 37 weeks. SGA is defined as a birth weight below the 10th percentile for gestational age. The time trend is defined as a continuous variable per year with 2003 as reference. Possible deviations from the time trend are defined as interactions between time dummies (reference 2003, per year) * intervention. The total number of observations is 542,824 for perinatal mortality and 539,909 for SGA and preterm birth.

	Perinatal mortality			SGA			Preterm birth		
independent variables	OR	95% CI		OR	95% CI		OR	95% CI	
Time (years, 2003-2009, reference 2003, continuous variable)	0.942	0.926	0.959	0.975	0.970	0.979	1.004	0.998	1.010
difference intervention and control	0.654	0.452	0.948	1.184	1.086	1.291	0.976	0.865	1.101
*interaction time (dummy)*intervention (reference 2003)*									
2004	1.127	0.672	1.889	0.933	0.825	1.056	1.195	1.015	1.407
2005	1.620	1.002	2.621	0.958	0.846	1.084	1.069	0.905	1.263
2006	1.158	0.679	1.975	0.980	0.865	1.111	1.203	1.021	1.419
2007	1.938	1.193	3.148	0.910	0.799	1.037	1.060	0.893	1.257
2008	1.376	0.812	2.334	0.999	0.879	1.134	1.149	0.973	1.357
2009	1.272	0.740	2.185	1.018	0.897	1.155	1.064	0.900	1.258
poverty	1.318	1.218	1.425	1.432	1.400	1.464	1.189	1.157	1.223
dutch	0.783	0.728	0.842	0.794	0.778	0.811	0.985	0.960	1.010
parity (n=2)	0.737	0.684	0.794	0.533	0.522	0.544	0.606	0.591	0.621
parity (n=3+)	1.292	1.133	1.473	0.492	0.467	0.517	0.804	0.762	0.848
age 25-34	0.893	0.809	0.987	0.845	0.823	0.868	0.971	0.939	1.005
age >=35	1.179	1.049	1.325	0.912	0.882	0.943	1.055	1.012	1.099
