# Supplementary material for: Did an urban perinatal health programme in Rotterdam, the Netherlands, reduce adverse perinatal outcomes? Register-based retrospective cohort study
Source: BMJ Open. 2019 Oct 22;9(10):e031357. doi: 10.1136/bmjopen-2019-031357 (PMC6830581; doi:10.1136/bmjopen-2019-031357)
Supplement: Supplementary data [file bmjopen-2019-031357supp005.pdf]

Supplementary file 5: the number and percentage of low SES pregnant women that are poor by year and the threshold of disposable household income that defines poverty.

Year	No. of deliveries				No. of low SES deliveries				% low SES deliveries			
	Total	NL - other	Control	Intervention	Total	NL - other	Control	Intervention	Total	NL-other	Control	Intervention
2003	187,578	99,171	82,431	5,976	37,749	15,196	19,858	2,695	20%	15%	24%	45%
2004	178,146	93,027	79,504	5,615	35,847	14,253	19,138	2,456	20%	15%	24%	44%
2005	175,231	90,968	78,604	5,659	35,238	13,793	18,983	2,462	20%	15%	24%	44%
2006	174,501	90,425	78,608	5,468	35,080	13,575	19,103	2,401	20%	15%	24%	44%
2007	171,618	88,861	77,465	5,292	34,482	13,275	18,901	2,306	20%	15%	24%	44%
2008	175,762	90,093	80,095	5,574	35,342	13,731	19,335	2,276	20%	15%	24%	41%
2009	178,213	90,717	81,587	5,909	35,789	13,981	19,408	2,401	20%	15%	24%	41%
2010	175,533	88,461	81,501	5,571	35,227	13,623	19,310	2,294	20%	15%	24%	41%
2011	175,346	87,821	81,488	6,037	35,189	13,283	19,357	2,549	20%	15%	24%	42%
2012	172,888	85,136	81,474	6,278	34,695	12,561	19,541	2,593	20%	15%	24%	41%
2013	166,248	81,786	78,553	5,909	33,389	12,331	18,672	2,387	20%	15%	24%	40%
2014	170,837	83,752	81,056	6,029	34,276	12,540	19,372	2,364	20%	15%	24%	39%
