# Supplementary material for: Did an urban perinatal health programme in Rotterdam, the Netherlands, reduce adverse perinatal outcomes? Register-based retrospective cohort study
Source: BMJ Open. 2019 Oct 22;9(10):e031357. doi: 10.1136/bmjopen-2019-031357 (PMC6830581; doi:10.1136/bmjopen-2019-031357)
Supplement: Supplementary data [file bmjopen-2019-031357supp006.pdf]

Supplementary file 6: Lagged treatment effect post intervention (dummies 2010-2014). Perinatal mortality is defined as still birth from 24 weeks onwards plus early neonatal mortality. Preterm is defined as born before a gestational age of 37 weeks. SGA is defined as a birth weight below the 10th percentile for gestational age. The total number of observations is 542,824 for perinatal mortality and 539,909 for SGA and preterm birth.

	Perinatal mortality			SGA			Preterm birth		
independent variables	OR	95% CI		OR	95% CI		OR	95% CI	
difference intervention and control before intervention	0.874	0.761	1.004	1.143	1.103	1.185	1.079	1.031	1.129
change in control area year 1 post intervention (2010)	0.807	0.725	0.897	0.911	0.885	0.938	1.017	0.984	1.051
change in control area year 2 post intervention (2011)	0.716	0.640	0.802	0.898	0.873	0.925	0.973	0.940	1.006
change in control area year 3 post intervention (2012)	0.758	0.679	0.847	0.876	0.851	0.902	0.944	0.912	0.976
change in control area year 4 post intervention (2013)	0.650	0.577	0.733	0.897	0.871	0.924	0.977	0.944	1.011
change in control area year 5 post intervention (2014)	0.608	0.538	0.687	0.891	0.865	0.917	0.957	0.925	0.991
intervention effect year 1 post intervention	1.169	0.786	1.740	0.955	0.860	1.060	1.081	0.956	1.221
intervention effect year 2 post intervention	0.724	0.442	1.188	0.983	0.888	1.087	1.020	0.902	1.153
intervention effect year 3 post intervention	1.074	0.717	1.608	0.925	0.835	1.025	0.932	0.821	1.058
intervention effect year 4 post intervention	1.254	0.825	1.905	0.964	0.870	1.068	0.920	0.809	1.047
intervention effect year 5 post intervention	1.465	0.980	2.189	0.947	0.854	1.050	0.956	0.842	1.086
poverty	1.377	1.292	1.467	1.442	1.417	1.467	1.202	1.176	1.228
dutch	0.790	0.745	0.838	0.774	0.762	0.787	0.993	0.974	1.012
parity (n=2)	0.756	0.712	0.802	0.534	0.525	0.543	0.612	0.600	0.623
parity (n=3+)	1.284	1.156	1.427	0.487	0.469	0.506	0.822	0.790	0.856
age 25-34	0.937	0.862	1.018	0.854	0.836	0.872	0.979	0.953	1.005
age >=35	1.214	1.103	1.337	0.931	0.907	0.956	1.076	1.042	1.111
