# Supplementary material for: Did an urban perinatal health programme in Rotterdam, the Netherlands, reduce adverse perinatal outcomes? Register-based retrospective cohort study
Source: BMJ Open. 2019 Oct 22;9(10):e031357. doi: 10.1136/bmjopen-2019-031357 (PMC6830581; doi:10.1136/bmjopen-2019-031357)
Supplement: Supplementary data [file bmjopen-2019-031357supp007.pdf]

supplementary file 7: Propensity score matching. Perinatal mortality is defined as still birth from 24 weeks onwards plus early neonatal mortality. Preterm is defined as born before a gestational age of 37 weeks. SGA is defined as a birth weight below the 10th percentile for gestational age. The total number of observations is 122,766 for perinatal mortality and 122,133 for SGA and preterm birth.

| | Perinatal mortality | | | SGA | | | Preterm birth | | |
|---|---|---|---|---|---|---|---|---|---|
| independent variables | OR | 95% CI | | OR | 95% CI | | OR | 95% CI | |
| linear time | 0.970 | 0.945 | 0.995 | 0.983 | 0.975 | 0.990 | 0.997 | 0.988 | 1.006 |
| difference intervention and control | 0.920 | 0.775 | 1.091 | 1.254 | 1.197 | 1.312 | 1.207 | 1.140 | 1.279 |
| intervention effect - slope change | 1.003 | 0.929 | 1.084 | 0.990 | 0.970 | 1.010 | 0.982 | 0.958 | 1.006 |
| poverty | 1.287 | 1.103 | 1.502 | 1.316 | 1.263 | 1.372 | 1.239 | 1.176 | 1.305 |
| dutch | 0.654 | 0.544 | 0.786 | 0.718 | 0.686 | 0.752 | 1.020 | 0.965 | 1.078 |
| parity (n=2) | 0.876 | 0.744 | 1.032 | 0.577 | 0.553 | 0.603 | 0.762 | 0.722 | 0.804 |
| parity (n=3+) | 1.353 | 1.056 | 1.733 | 0.489 | 0.449 | 0.533 | 0.989 | 0.902 | 1.084 |
| age 25-34 | 0.985 | 0.811 | 1.195 | 0.845 | 0.805 | 0.886 | 0.898 | 0.844 | 0.956 |
| age >=35 | 1.132 | 0.885 | 1.448 | 0.858 | 0.803 | 0.917 | 0.965 | 0.889 | 1.047 |
