# Supplementary material for: Did an urban perinatal health programme in Rotterdam, the Netherlands, reduce adverse perinatal outcomes? Register-based retrospective cohort study
Source: BMJ Open. 2019 Oct 22;9(10):e031357. doi: 10.1136/bmjopen-2019-031357 (PMC6830581; doi:10.1136/bmjopen-2019-031357)
Supplement: Supplementary data [file bmjopen-2019-031357supp008.pdf]

supplementary file 8: The intervention effect (slope change per year) of three interventions in selected boroughs (peer education, care pathways and birth centre) compared to the control population, using the same difference-in-difference model as in the main analysis. Perinatal mortality is defined as still birth from 24 weeks onwards plus early neonatal mortality. Preterm is defined as born before a gestational age of 37 weeks. SGA is defined as a birth weight below the 10th percentile for gestational age. The analyses are adjusted for time (year), difference between intervention and control group at t=0 (2003), age, parity, migration background and household income.

	perinatal mortality			SGA			preterm birth		
	OR	*95% CI*		*beta*	*95% CI*		*OR*	*95% CI*	
peer education	1.1826	1.0393	1.3457	0.9825	0.9489	1.0171	0.9634	0.9226	1.0059
care pathways	1.1165	0.9512	1.3104	0.9951	0.9552	1.0366	0.9855	0.9364	1.0372
birth centre	1.0583	0.9606	1.1659	0.9896	0.7054	1.0139	0.9706	0.9414	1.0008
