# Supplementary material for: Did an urban perinatal health programme in Rotterdam, the Netherlands, reduce adverse perinatal outcomes? Register-based retrospective cohort study
Source: BMJ Open. 2019 Oct 22;9(10):e031357. doi: 10.1136/bmjopen-2019-031357 (PMC6830581; doi:10.1136/bmjopen-2019-031357)

Supplementary file 1. Perinatal mortality by year for the control (blue line) and intervention area (red line). Perinatal mortality is defined as still birth from 24 weeks onwards plus early neonatal mortality per 1,000 births.

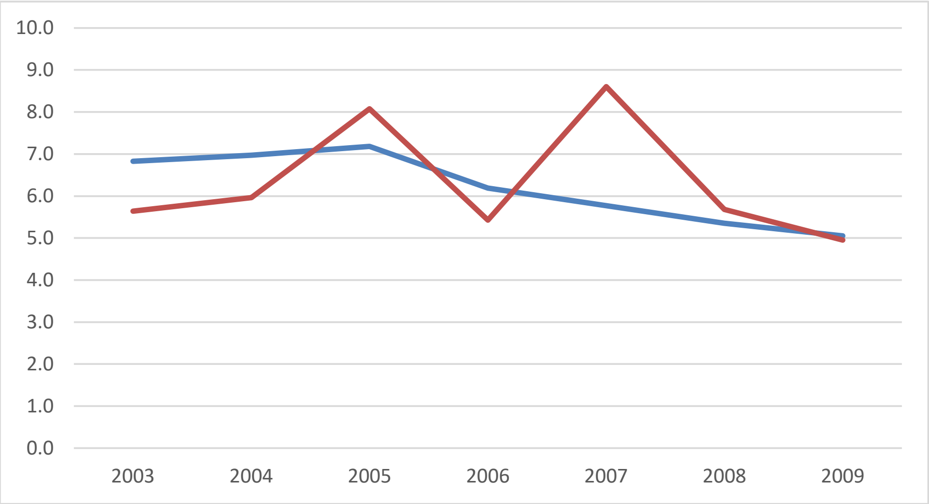

Supplement: Supplementary data [file bmjopen-2019-031357supp001.pdf]
