# Supplementary material for: Did an urban perinatal health programme in Rotterdam, the Netherlands, reduce adverse perinatal outcomes? Register-based retrospective cohort study
Source: BMJ Open. 2019 Oct 22;9(10):e031357. doi: 10.1136/bmjopen-2019-031357 (PMC6830581; doi:10.1136/bmjopen-2019-031357)

Supplementary file 2. Preterm birth by year for the control (blue line) and intervention area (orange line). Preterm birth is defined as born before a gestational age of 37 weeks per 1,000 live births.

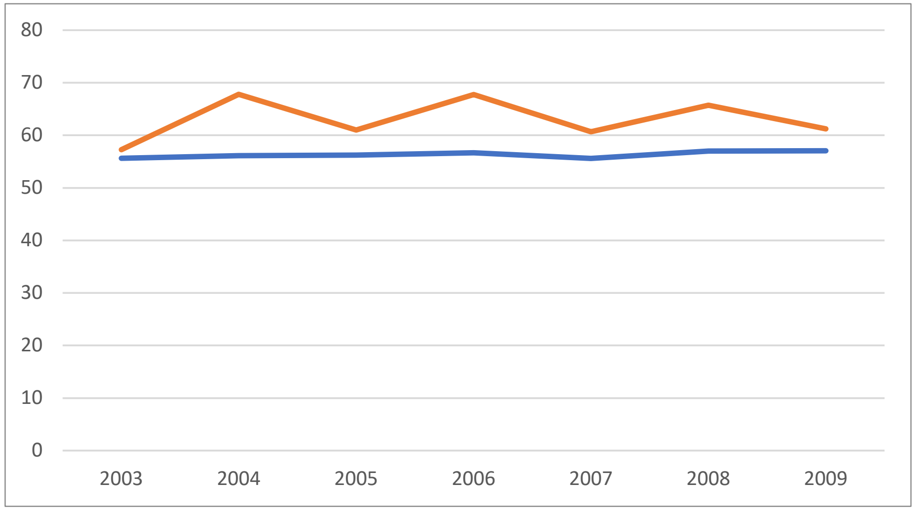

Supplement: Supplementary data [file bmjopen-2019-031357supp002.pdf]
