# Supplementary material for: Did an urban perinatal health programme in Rotterdam, the Netherlands, reduce adverse perinatal outcomes? Register-based retrospective cohort study
Source: BMJ Open. 2019 Oct 22;9(10):e031357. doi: 10.1136/bmjopen-2019-031357 (PMC6830581; doi:10.1136/bmjopen-2019-031357)
Supplement: Author's manuscript [file bmjopen-2019-031357.draft_revisions.pdf]

BMJ Open

BMJ Open is committed to open peer review. As part of this commitment we make the peer review history of every article we publish publicly available.

When an article is published we post the peer reviewers' comments and the authors' responses online. We also post the versions of the paper that were used during peer review. These are the versions that the peer review comments apply to.

The versions of the paper that follow are the versions that were submitted during the peer review process. They are not the versions of record or the final published versions. They should not be cited or distributed as the published version of this manuscript.

BMJ Open is an open access journal and the full, final, typeset and author-corrected version of record of the manuscript is available on our site with no access controls, subscription charges or pay-per-view fees (http://bmjopen.bmj.com).

If you have any questions on BMJ Open's open peer review process please email info.bmjopen@bmj.com

BMJ Open

The effects of an urban perinatal health programme on perinatal outcomes: a difference-in-differences analysis.

Journal:	*BMJ Open*
Manuscript ID	bmjopen-2019-031357
Article Type:	Research
Date Submitted by the Author:	29-Apr-2019

Complete List of Authors:	de Jonge, Hendrik C. C.; Erasmus MC, Public Health<br>Lagendijk, Jacqueline; Erasmus MC, Department of Obstetrics and Gynaecology<br>Saha, Unnati; Erasmus MC, Public Health<br>Been, JV; Erasmus University Medical Centre–Sophia Children’s Hospital, Paediatrics<br>Burdorf, Alex; Erasmus MC, Department of Public Health

Keywords:	PUBLIC HEALTH, EPIDEMIOLOGY, PERINATOLOGY

SCHOLARONE™
Manuscripts

The effects of an urban perinatal health programme on perinatal outcomes: a difference-in-differences analysis.

Erik de Jonge (h.c.c.dejonge@erasmusmc.nl) [1*]

Jacqueline Lagendijk (j.lagendijk.2@erasmusmc.nl) [2]

Unnati Rani Saha (u.saha@erasmusmc.nl) [1]

Jasper V. Been (j.been@erasmusmc.nl) [1,2,3]

Alex Burdorf (a.burdorf@erasmusmc.nl) [1]

Affiliations:

1. Erasmus MC, University Medical Center Rotterdam, Department of Public Health

2. Erasmus MC, University Medical Centre Rotterdam, Department of Obstetrics and Gynaecology

3. Erasmus MC - Sophia Children's Hospital, University Medical Center Rotterdam, Department of Pediatrics, Division of Neonatology

Corresponding author:

Erik de Jonge, Department of Public Health, Erasmus MC, P.O. Box 2040, 3000 CA Rotterdam, the Netherlands.
Email: h.c.c.dejonge@erasmusmc.nl

Number of words (excl. title page, abstract and literature): 3,707 words.

For peer review only

For peer review only - http://bmjopen.bmj.com/site/about/guidelines.xhtml

Abstract

Introduction

In Rotterdam, the second largest city in the Netherlands, perinatal mortality is higher than the national average, most notably in its deprived areas. An urban perinatal health programme consisting of health promotion, risk selection and risk-guided pregnancy care, and a new primary care child birth centre was implemented 2009-2012 in Rotterdam. We studied the effects of the programme on perinatal outcomes using a difference-in-differences (DiD) analysis.

Methods

We used individual-level perinatal outcome data from the Dutch Perinatal Registry 2003-2014 linked to Central Bureau of Statistics data with individual disposable household income. We compared trends in perinatal mortality, preterm delivery, and small for gestational age births between targeted urban neighbourhoods in Rotterdam (intervention) and all other urban neighbourhoods in the Netherlands (control). The effect of the programme was modelled as a change in trend of each perinatal outcome in the treatment group post-intervention from January 2010 onwards compared to the control population. All analyses were adjusted for maternal age, parity, ethnicity, and low socio-economic status (SES). We also conducted a stratified analysis by SES.

Results

During 2003-2014 downward trends in perinatal mortality (adjusted odds ratio (aOR) 0.94 per year, 95% confidence interval (CI) 0.94-0.95), small for gestational age births (aOR 1.00 per year, 95% CI 0.99-1.00), and preterm birth (aOR 0.98 per year, 95% CI 0.98-0.98) in the entire study population were observed. No demonstrable changes in trends in these perinatal outcomes were found post-intervention in the intervention group. The stratified analysis by SES showed no changes in trends post-intervention in both strata.

Discussion

The urban perinatal health programme had no demonstrable effects on perinatal outcomes. The municipal intervention may not have reached a sufficient proportion of the population or has provided too little contrast to the widespread attention for inequalities in pregnancy outcomes occurring simultaneously in the Netherlands.

For peer review only - http://bmjopen.bmj.com/site/about/guidelines.xhtml

Strengths & limitations

- The nationally representative sample of almost a million pregnancies during the period 2003-2014 had a good discriminatory power to estimate the effect of the programme on a rare outcome like perinatal mortality.
- The linkage across three routinely collected data registries provided pregnancy outcomes and socioeconomic data, allowing adjustment for important medical and social determinants in the statistical analysis.
- We applied difference-in-differences analysis, an econometric method, that allows us to determine programme effects on population level.
- All pregnant women in the urban neighbourhoods targeted with the intervention were considered to be exposed to the intervention, whether or not these women were actually reached by activities within the complex intervention, which may have attenuated observed associations.
- The relative good performance of the urban perinatal health care system in Rotterdam before the intervention period and unobserved improvements in urban perinatal health care during the intervention period in the Netherlands may have attenuated the observed intervention effect.

For peer review only

Introduction

In the 2000s the Netherlands had a relatively high perinatal mortality, ranking third highest among 26 European countries in 2004 and sixth highest in 2010 (1-3). Considerable regional inequalities in perinatal outcomes were reported within the Netherlands (4). In Rotterdam, the second largest city in the Netherlands, perinatal mortality was markedly higher than nationally, most notably in its deprived neighbourhoods (5). In a child-birth cohort study, these neighbourhood differences in various perinatal outcomes could largely be attributed to the increased prevalence of medical, as well as, social risk factors (6).

In response to these findings, the Erasmus MC in collaboration with the Municipal Health Service Rotterdam-Rijnmond initiated an urban perinatal health programme, called "Ready for a Baby", with the aim to improve perinatal outcomes in Rotterdam. The programme consisted of several intervention components across the pregnancy care chain: preconception health promotion, improved risk selection and risk guided care during pregnancy, and the establishment of a primary care birth centre. These components were gradually introduced in the period 2009-2012, and, depending on the component, reached nearly city wide coverage (preconception health promotion) or only specific neighbourhoods (e.g. primary care birth centre) (7).

Perinatal mortality in the Netherlands has gradually reduced over the past two decades. Favourable trends in risk factors may have contributed, including a reduction in smoking by pregnant women, less multiple births, increased use of ultrasonography at 20 weeks gestation for detection of congenital abnormalities, and improved care for very premature babies (8-9). The introduction of the "Ready for a Baby" programme in Rotterdam was conceptualised as a natural experiment (10). In order to evaluate whether this programme has had an additional impact on the secular trends of decline in unfavourable perinatal outcomes, the Difference-in-Differences (DiD) method was considered the appropriate approach to evaluate the effects of an intervention in an observational study. In this method the change in health in the intervention group before and after the introduction of the intervention (difference) can be distinguished from changes in health over time in both the intervention and control groups (differences) (11). Therefore, the aim of this study was to determine the influence of the urban perinatal health programme "Ready for a Baby" on adverse perinatal health outcomes.

For peer review only - http://bmjopen.bmj.com/site/about/guidelines.xhtml

Methods

Study design and population

Routinely collected birth data from the Dutch Perinatal registry, enriched with national registers with personal information, were used for a DiD analysis in the period 2003-2014. The start in year 2003 was determined by individual information on socio-economic status (SES) becoming available in national registers. The year 2014 was the last year with complete information available at time of this study. The study population consisted of all singleton deliveries in urban neighbourhoods in the Netherlands during the study period, comprising approximately 45% of all deliveries in the Netherlands. A neighbourhood is defined by postal code and an urban neighbourhood has more than 1500 houses per square kilometre (12). The intervention group consisted of all deliveries in 51 urban neighbourhoods in Rotterdam, where at some point in time during the intervention period a component of the urban perinatal health programme was implemented. The control group comprised all deliveries in other urban postal code areas in the Netherlands including six untargeted urban neighbourhoods in Rotterdam.

Programme description

The "Ready for a Baby" programme had three components: health promotion in preconception care, improved risk selection and risk guided care, and establishment of a primary care birth centre in the university medical centre. The content of the programme has been published in detail before and will be described here briefly (7).

The first component of the programme aimed to promote preconception health by three strategies. The first strategy aimed to collectively increase awareness of the importance of preconception health through mass media campaigns (including flyers, posters, editorials, and advertisements in local Dutch and Turkish newspapers, on busses and trams, at offices of health care providers, pharmacists, retailers, and at churches and mosques). Besides increasing awareness, these campaigns aimed to promote favourable attitudes and behaviours for a healthy pregnancy, such as the use of folic acid and the cessation of smoking and alcohol use. This strategy targeted all citizens in Rotterdam and was not confined to the intervention group. The second strategy used peer education to increase preconception-related health literacy and motivation to attend preconception care consultations, especially amongst low SES and migrant groups (13). In targeted neighbourhoods six women and one man were trained as peer educators. These peer educators then recruited about 2,300 participants during the course of the programme for peer education group sessions through their existing network and community meeting places (e.g. mosques and schools). The sessions were interactive, often in multiple languages, and provided participants information on the influence of lifestyle changes on pregnancy outcomes, and additionally advised on where to obtain individual consultations. The third strategy in targeted neighbourhoods was the provision of individual preconception care consultations by general practitioners (GPs) and community midwives. At least 43 couples attended an individual consultation after

For peer review only - http://bmjopen.bmj.com/site/about/guidelines.xhtml

receiving tailored health promotion from a web-based preconception health assessment "Preconceptiewijzer.nl" (13-14).

The second component of the programme aimed to improve risk selection and risk guided care by use of the Rotterdam Reproductive Risk Reduction (R4U) scorecard along with the Shared-Care model (15-17) in the targeted neighbourhoods. Since midwives are usually the first point of contact of a pregnant woman, the vast majority (n=46) of all community midwives in target neighbourhoods in Rotterdam were trained to use and integrate the R4U scorecard along with the Shared-Care model into their daily practice. The R4U scorecard is a systematic risk assessment in the first trimester of pregnancy focussing on medical and non-medical risk factors related to adverse pregnancy outcomes, including for example migration background and low household income (16-17). The Shared-Care model is an approach to risk guided care that has three elements: 1) continuity of care (e.g. a case manager is assigned to high risk women who need care from different professionals, care pathways are defined for risks identified in the R4U), 2) patient centeredness (e.g. through fostering of self-management, and efforts to combine appointments to different care providers), and 3) interprofessional collaboration (e.g. through formulating a joint set of aims and ambitions for collaboration including care pathways, training in team work, and interprofessional education) (15). The R4U-scorecard guided the care pathways in the Shared-Care model through templates describing the consecutive steps a professional was advised to take to reduce the potential contribution of identified risk factors for adverse perinatal health outcomes. In order to enhance the efficiency and quality of the local antenatal health care, all details of instruments and templates were discussed during meetings with community midwives, obstetricians, and social workers under guidance of a member of the "Ready for a Baby" programme.

The third component of the programme was the establishment of a primary care birth centre (PCBC) in the Erasmus University Medical Centre in Rotterdam. The PCBC is a separate facility led by community midwives, where women can deliver at their own discretion if a hospital delivery is not medically indicated but social factors make a home delivery less preferable (18-19). The PCBC aimed to provide risk-directed care by assessing the risk status of each woman at different intervals (e.g. upon arrival at the PCBC, during labour, and postpartum).

Figure 1 shows how the different parts of the intervention were introduced from July 2009 until December 2012. The first component of the programme, preconception health promotion, targeted primarily women in selected neighbourhoods, but by nature of the instrument the mass media approach had a reach in all neighbourhoods in Rotterdam. The second component, the risk selection and shared care, was implemented in the selected neighbourhoods. The third component, the new birth centre, primarily served pregnant women from the community midwife practices around Erasmus MC. So the different components were introduced at different moments in time and in different neighbourhoods.

For peer review only - http://bmjopen.bmj.com/site/about/guidelines.xhtml

Data

We used data from the Dutch Perinatal Registry 2003-2014 (Perined, https://www.perined.nl). The Dutch Perinatal Registry is a registry with information provided by midwives, gynaecologists, paediatricians, and general practitioners and contains demographic characteristics, medical risk factors, obstetric history, and pregnancy and neonatal outcomes (20-21). Through individual-level linkage the Dutch Perinatal Registry was enriched with two nationwide registries from Statistics Netherlands. Household income from the Integral Household Income (IHI) register, based on tax information from 2003 onwards (22), was used to define low socio-economic status by lowest 20% disposable household income. The second, the Dutch population register, provided information on migration background (23).

A trusted third party (an in-house service at Statistics Netherlands) merged these datasets using four-digit postal code, birth date of the mother and birth date of the child after which the identifying variables were removed. The merged one-way coded dataset was made available in a secure research environment at Statistics Netherlands for analysis. Results were rigorously checked for identifiability by Statistics the Netherlands before they were released from the secure research environment for publication. This procedure is in accordance with Dutch legislation and the Dutch Code of Conduct for Medical Research for use of anonymous data for research purposes without an explicit informed consent (24).

Deliveries with less than 24 completed weeks of gestation from the dataset were excluded, because most of these deliveries will end in stillbirth. These stillbirths aren't registered in the population registry according to Dutch law so can't be linked to household income and migration background data, which were required for the statistical analysis. Records with other missing covariates for the statistical analysis were also excluded.

Outcomes

Our primary outcomes were: perinatal mortality, preterm birth, and small for gestational age (SGA) birth. Perinatal mortality was defined as the number of stillbirths from 24 completed weeks of gestation or early neonatal deaths (i.e. death of a liveborn baby within the first seven days of life) per 1,000 singleton births. Preterm birth was defined as birth before 37 completed weeks of gestation per 1,000 singleton livebirths. SGA was defined as any baby that was smaller than the 10th centile, corrected for gestational age in weeks and sex, according to the Visser curve, per 1,000 singleton livebirths (25).

Statistical analysis

A DiD analysis was conducted with logistic regression models for perinatal mortality, preterm birth, and SGA as dichotomous dependent variables.

For peer review only - http://bmjopen.bmj.com/site/about/guidelines.xhtml

Each model has three key independent variables and several confounders. The first variable is the year of birth as continuous variable, which captures the trend over time and is expressed by the odds ratio (OR) for trend per year that represents the yearly increase or decrease in likelihood of perinatal outcome. The second variable is a dummy variable with value 1 for the intervention group and value 0 for the control group. This dummy variable reflects differences in perinatal health between intervention and control groups at baseline. The third variable is an interaction term between years since start of the programme as continuous variable and intervention group status. This term corresponds to the DiD estimate, as it presents differences in perinatal health trends between intervention and control groups post-intervention over and above the underlying temporal trends (15). It is important to note that this DiD estimate presents the change in slope of the trend for the intervention group post-intervention. Thus, we did not model the invention as an immediate effect (step function) that introduces a constant difference between intervention and control group from start of the programme until 2014. The programme was gradually introduced from July 2009 onwards and, given the duration of pregnancy, we assumed that the intervention could take effect first from 2010 onwards. Therefore, a DiD analysis on change in the trend per year in the intervention group after 2010 was considered most appropriate.

A crucial assumption in the DiD analysis is the parallel trend assumption (15), i.e. that pre-intervention trends were similar in intervention and control groups over the period 2003-2009. This assumption was tested by an interaction between the dummy variable and year of birth between 2003-2009, which indicates whether the baseline difference between intervention and control group changed per year. This analysis showed that the parallel trend assumption was not violated for any of the perinatal outcomes.

We included four major risk factors that were targeted in the intervention (SES, ethnicity, parity and age) as confounders. This step was taken to improve the comparability of the intervention and control groups in the analysis. SES is associated with perinatal outcomes and was differently distributed in the intervention and control groups, therefore lowest 20 percentile household income was used as indicator of SES. As the intervention targeted particularly low-SES women, we also conducted stratified analyses according to SES. Ethnicity is associated with perinatal outcomes and with urbanisation, and hence was included as confounder. Non-Dutch ethnicity was defined as any person who was born in another country than the Netherlands (first generation migration background) or had at least one parent born in another country (second generation migration background). We also included parity and age at delivery as potential confounders.

Patient and public involvement

This research was done without patient or public involvement. However, the research question was proposed by the Municipal Health Service Rotterdam as part of their legal task of monitoring population health (Dutch Public Health Law).

For peer review only - http://bmjopen.bmj.com/site/about/guidelines.xhtml

Results

Study population

From 2003-2014, a total of 2,116,226 deliveries was available in the Dutch Perinatal Registry, encompassing 96.6% of all deliveries in the Netherlands in that period [20-21]. A total of 1,031,683 deliveries was registered within an urban neighbourhood of the intervention and control group, thus representing 49% of all deliveries in the Netherlands (figure 2). We excluded all deliveries before 24 weeks of completed gestation and all multiple births, which were 38,931 deliveries (3.8% of all deliveries) of which 3,704 perinatal deaths (39% of all perinatal deaths (Supplementary File 1)). In addition, we excluded deliveries that could not be matched to Statistics Netherlands data (household income or migration background)(16,319 deliveries, 1.6%) or lacked information on any other confounder (33,819 deliveries, 3.3%). Thus, the total study population comprised 942,614 deliveries (figure 2).

In the intervention group 83% of all deliveries took place in deprived urban neighbourhoods, whereas in the control group the corresponding figure was 39% (table 1). Women delivering in the intervention group were more often younger, multiparous, non-Dutch, and more often had a household income below the 20th percentile. The percentage of women with more than one of these risk factor was considerably higher in the intervention population (59%) than in the control population (34%). In addition, in deprived neighbourhoods in the intervention group pregnant women had a higher prevalence of risk factors than pregnant women in deprived neighbourhoods of the control group, specifically non-Dutch background (73% vs. 49%) and low dispensable household income (46% vs. 35%). The incidence rates for perinatal mortality, preterm birth and for SGA were higher in the intervention group than in the control group, both in deprived as well as non-deprived neighbourhoods.

For peer review only

Table 1: Socio-demographic characteristics, presence of risk factors, and perinatal mortality, preterm birth, and small-for-gestational age over the period 2003-2014 in intervention group (targeted urban neighbourhoods in Rotterdam) and control group (other urban neighbourhoods in the Netherlands), stratified by deprivation of postal code area.*

	Intervention area						Control areas					
	All		*Deprived*		*Non-deprived*		*All*		*Deprived*		*Non-deprived*	
Total no. of deliveries		61,415		50,727		10,688		881,202		340,914		540,288
Parity												
Nulliparous	48%	29,308	46%	23,180	57%	6,128	47%	418,074	47%	160,270	48%	257,804
1-2	44%	26,880	45%	22,716	39%	4,164	47%	417,538	46%	156,892	48%	260,646
3+	9%	5,227	10%	4,831	4%	396	5%	45,590	7%	23,752	4%	21,838
Ethnicity (non Dutch)	68%	41,702	73%	36,951	44%	4,751	37%	321,950	49%	167,943	29%	154,007
Age of mother												
<25	20%	12,423	22%	11,319	10%	1,104	13%	110,465	18%	60,191	9%	50,274
25-29	30%	18,361	31%	15,679	25%	2,682	28%	250,322	31%	105,834	27%	144,488
30-34	31%	18,917	29%	14,522	41%	4,395	37%	327,361	33%	111,349	40%	216,012
35-39	16%	9,616	15%	7,491	20%	2,125	19%	164,403	16%	53,252	21%	111,151
>=40	3%	2,098	3%	1,716	4%	382	3%	28,651	3%	10,288	3%	18,363
Socio-economic status												
lowest 20% disposable household income	42%	25,919	46%	23,431	23%	2,488	24%	212,405	35%	120,488	17%	91,917
More than 1 risk factor†	59%	36,139	63%	29,638	43%	6,501	34%	299,727	49%	129,418	27%	170,309
Perinatal mortality (/1,000 stillbirths & live singleton births)												

For peer review only

For peer review only - http://bmjopen.bmj.com/site/about/guidelines.xhtml

2003-2009	6.5	223	6.7	202	4.8	21	6.2	3,173	7.1	1,436	5.7	1,737
2010-2014	5.1	136	5.7	116	3.2	20	4.5	1,667	5.2	725	4.0	942
Preterm birth rate (/1,000 live singleton births)												
2003-2009	63	2,166	64	1,917	57	249	56	28447	60	12,066	54	16,381
2010-2014	60	1,611	61	1,254	57	357	55	20376	59	8,098	53	12,278
SGA rate (1,000 live singleton births)												
2003-2009	109	3,756	113	3,377	87	379	84	42,505	96	19,330	76	23,175
2010-2014	94	2,513	98	2,007	80	506	76	28,249	86	11,898	70	16,351

* Deprivation is defined as the lowest quintile of postal codes by status score, an area level composite index of individual household income, education and employment status calculated every four years by the Netherlands Institute of Social Research.

† The following risk factors are considered: nulliparous, non Dutch ethnicity, age of the mother <25 year, lowest 20% disposable household income

For peer review only

For peer review only - http://bmjopen.bmj.com/site/about/guidelines.xhtml

Differences-in-differences analysis

Table 2 shows that the likelihood of perinatal mortality decreased during 2003-2014 by about 6% per year across all urban neighbourhoods in the Netherlands (adjusted odd ratio (aOR) 0.9439/year (95% confidence interval (CI) 0.9362-0.9517)). SGA decreased by 2% per year (aOR 0.9809 (95% CI 0.9787-0.9831)) and preterm birth decreased by 0.3% per year (aOR 0.9970 (95% CI 0.9944-0.9997)). Small differences in annual trends between the intervention and the control group were observed, albeit not statistically significant. The DiD analysis on the effect of the urban perinatal health programme showed that during the post-intervention period in the intervention group perinatal mortality (aOR 1.0535 (95% CI 0.9889-1.1223) increased slightly each year, whereas preterm birth (aOR 0.9809 (95% CI 0.9619-1.0004) and SGA (aOR 0.9928 (95% CI 0.9772-1.0086)) showed modest improvements.

The analysis showed that for the pre-intervention period, after adjustment for important confounders, perinatal mortality was 14% lower in the intervention population than in the control population (aOR 0.8601, 95% CI 0.7534-0.9819). Preterm birth (aOR 1.0962 (95% CI 1.0505-1.1440)) and SGA (aOR 1.1313 (95% CI 1.0935-1.1703)) were higher in the intervention population than in the control population.

For peer review only

For peer review only - http://bmjopen.bmj.com/site/about/guidelines.xhtml

Table 2. Logistic regression models with difference-in-differences analyses of the effect of the urban perinatal health programme on perinatal mortality, preterm birth and SGA.

	Perinatal mortality			Preterm birth			SGA		
independent variables	OR	95% CI		OR	95% CI		OR	95% CI	
trend per year	0.9439	0.9362	0.9517	0.9970	0.9944	0.9997	0.9809	0.9787	0.9831
difference intervention and control group	0.8601	0.7534	0.9819	1.0962	1.0505	1.1440	1.1313	1.0935	1.1703
programme effect (change in trend per year from 2010 onwards)	1.0535	0.9889	1.1223	0.9809	0.9619	1.0004	0.9928	0.9772	1.0086
low SES	1.3761	1.2917	1.4660	1.2017	1.1764	1.2275	1.4417	1.4170	1.4668
ethnicity non Dutch	1.2690	1.3462	1.1963	1.0068	1.0264	0.9876	1.2927	1.3134	1.2722
parity 0 (reference)	1.0000			1.0000			1.0000		
parity 1-2	0.7560	0.7120	0.8026	0.6114	0.5998	0.6232	0.5340	0.5254	0.5427
parity 3+	1.2823	1.1541	1.4248	0.8218	0.7894	0.8554	0.4871	0.4687	0.5062
age <25 (reference)	1.0000			1.0000			1.0000		
age 25-34	0.9401	0.8654	1.0212	0.9786	0.9529	1.0050	0.8554	0.8377	0.8735
age >=35	1.2238	1.1112	1.3479	1.0762	1.0423	1.1111	0.9339	0.9100	0.9585
baseline rate*	8.6	7.8	9.4	71	69	74	155	151	159

Perinatal mortality is defined as still birth from 24 weeks onwards plus early neonatal mortality. The total number of observations for the perinatal mortality model is 942,614. Preterm is defined as born before a gestational age of 37 weeks and SGA is defined as a birth weight below the 10th percentile corrected for gestational age and sex. The total number of observations for the preterm birth and SGA models is 937,639.

* for perinatal mortality per 1,000 live and stillbirths, for preterm birth and SGA per 1,000 live births

For peer review only

The stratified analysis by socio-economic status of the pregnant woman showed very similar results for pregnant women with low SES and pregnant women with higher SES. The intervention had no demonstrable influence on trends in any of the perinatal outcomes post-intervention (table 3).

For peer review only

For peer review only - http://bmjopen.bmj.com/site/about/guidelines.xhtml

Table 3. Stratified analysis by socio-economic status of the mother with logistic regression models with difference-in-differences analyses of the effect of the urban perinatal health programme on perinatal mortality, preterm birth and SGA.

**Lowest quintile household income**	Perinatal mortality			Preterm birth			SGA		
independent variables	OR	95% CI		OR	95% CI		OR	95% CI	
trend per year	0.9516	0.9380	0.9654	1.0008	0.9958	1.0059	0.9864	0.9825	0.9903
difference intervention and control group	0.8209	0.6786	0.9929	1.1677	1.0957	1.2444	1.1296	1.0749	1.1871
programme effect (change in trend per year from 2010 onwards)	1.0673	0.9761	1.1670	0.9767	0.9481	1.0062	0.9947	0.9718	1.0181
ethnicity non Dutch	1.3668	1.5181	1.2307	0.8716	0.9024	0.8418	1.0167	1.0450	0.9893
parity 0 (reference)	1.0000			1.0000			1.0000		
parity 1-2	0.7392	0.6652	0.8214	0.7826	0.7545	0.8117	0.6258	0.6084	0.6438
parity 3	1.0544	0.8960	1.2407	1.0134	0.9539	1.0766	0.5605	0.5308	0.5918
age <25 (reference)	1.0000			1.0000			1.0000		
age 25-34	1.0666	0.9478	1.2004	0.9566	0.9188	0.9960	0.8914	0.8647	0.9190
age >=35	1.2893	1.1082	1.4999	1.1172	1.0597	1.1778	0.9334	0.8947	0.9737
baseline rate*	11.2	9.8	12.7	70	67	74	181	175	188

**Household income above 20th percentile**	Perinatal mortality			Preterm birth			SGA		
independent variables	OR	95% CI		OR	95% CI		OR	95% CI	
trend per year	0.9402	0.9308	0.9497	0.9961	0.9930	0.9992	0.9787	0.9761	0.9813
difference intervention and control group	0.9084	0.7553	1.0926	1.0472	0.9884	1.1094	1.1500	1.0978	1.2046
intervention effect (change in trend per year from 2010 onwards)	1.0294	0.9403	1.1270	0.9841	0.9585	1.0103	0.9876	0.9663	1.0093
ethnicity non Dutch	1.2108	1.3017	1.1262	1.0665	1.0913	1.0423	1.4399	1.4678	1.4126
parity 0 (reference)	1.0000			1.0000			1.0000		
parity 1-2	0.7592	0.7057	0.8166	0.5592	0.5467	0.5721	0.4956	0.4859	0.5056

For peer review only

For peer review only - http://bmjopen.bmj.com/site/about/guidelines.xhtml

parity 3+	1.4987	1.3058	1.7201	0.7298	0.6905	0.7713	0.4446	0.4206	0.4700
age <25 (reference)	1.0000			1.0000			1.0000		
age 25-34	0.8417	0.7489	0.9461	0.9544	0.9206	0.9894	0.8185	0.7950	0.8427
age >=35	1.1253	0.9880	1.2816	1.0446	1.0024	1.0886	0.9142	0.8835	0.9459
baseline rate*	9.2	8.1	10.4	80	76	83	179	173	185

Perinatal mortality is defined as still birth from 24 weeks onwards plus early neonatal mortality. The total number of observations for the perinatal mortality model is 238,226 for low SES and 704,388 for high SES. Preterm is defined as born before a gestational age of 37 weeks and SGA is defined as a birth weight below the 10th percentile corrected for gestational age and sex. The total number of observations for the preterm birth and SGA models is 236,737 for low SES and 701,524 for high SES. Low SES was defined as the lowest 20 percentile by disposable household income per year.

* for perinatal mortality per 1,000 live and stillbirths, for preterm birth and SGA per 1,000 live births

For peer review only

Discussion

In this study, the influence of an urban perinatal health programme in Rotterdam on perinatal health outcomes was evaluated by a DiD approach, an analytical technique for natural experiment evaluation in an observational setting. The DiD analysis could not demonstrate that the introduction of the programme influenced trends in perinatal mortality, preterm birth, or small-for-gestational age birth in the post-intervention years in the intervention group.

Strengths of this study include the large study population and the available information on important confounders. The nationally representative sample of almost a million pregnancies during the period 2003-2014 had a good discriminatory power to estimate the effect of the programme on a rare outcome like perinatal mortality. The linkage across three routinely collected data registries provided pregnancy outcomes and socioeconomic data, allowing adjustment for important medical and social determinants in the statistical analysis.

Our study also has several limitations pertaining to data availability, and to the DiD analysis. Availability of data was dictated by the registers used. SES is an important determinant of perinatal health (26), but in our analysis only disposable household income was available. Highest educational attainment based on certified diploma registers had a high percentage of missing values, and could therefore not be included as a covariate in our study. Using only a one-dimensional representation of SES might not fully adjust for residual differences between the intervention and the control group.

The DiD analysis applied in this study has several limitations. First, a crucial assumption is that trends in outcome in intervention and control groups in the years before the intervention are parallel, i.e. have a constant difference, captured in the DiD logistic regression model by the difference at baseline (table 2). Evaluation showed that this assumption was not violated. The DiD analysis accounts for time-invariant differences between the intervention and control groups, as well as any factors that equally change over time in both groups. The descriptive information showed that in the intervention neighbourhoods in Rotterdam there is much higher accumulation of risk factors among pregnant women, and that a much larger proportion of women lived in deprived neighbourhoods. In our analysis we adjusted for these risk factors to ensure comparability of intervention and control groups. However, it cannot be discarded that women in Rotterdam have experienced less favourable trends in other, unobserved, risk factors during the observation period of this study, which may have attenuated any potential effect of the intervention. Second, the DiD makes the strong assumption that the precise timing of the intervention is completely at random, creating exogenous variation that allows causal inference. This assumption cannot be formally tested, but it must be acknowledged that the urban perinatal health programme was designed and implemented in response to the relatively high perinatal mortality in the Netherlands and opportunities for improvement in prevention and child health care in Rotterdam.

At the start of the “Ready for a Baby” programme, adjusted for risk factors, perinatal mortality was lower in Rotterdam than in other urban areas in the Netherlands. This favourable position of Rotterdam might be partly

For peer review only - http://bmjopen.bmj.com/site/about/guidelines.xhtml

attributed to the large concentration of hospitals in Rotterdam and attention in the local child health care system for high-risk women since they constitute a relatively large part of all pregnant women. Therefore, the DiD analysis may have not been able to capture additional change in an already decreasing trend in perinatal mortality in the intervention group. A linked issue is that improvements in perinatal health care may have occurred also in other neighbourhoods. These co-interventions may have biased the comparisons between intervention and control groups.

It is important to consider that this study evaluates the possible influence of a population intervention rather than the effects of an intervention at individual level. The content of the complex intervention comprises universal primary prevention and changes in quality and delivery of child health care, which are notoriously difficult to evaluate at individual level. Also, the different components of the intervention were introduced gradually during the intervention period and had varying coverage: from nearly city wide to certain neighbourhoods only. In the analysis, all pregnant women in the targeted urban neighbourhoods with the intervention were considered to be exposed to the intervention, whether or not these women were actually reached by activities within the complex intervention. This might have attenuated observed associations, when a substantial number of women would not have been included in components of the programme. For some parts of the programme, additional information was collected. The coverage of the preconception health peer education program was probably at best 15%, given that about 5,000 women deliver annually in the targeted neighbourhoods in Rotterdam and 2,300 women were reached during a three year period. In contrast, the majority of midwifes was trained to use the R4U scorecard and Shared Care method, resulting in a large uptake of the second component of the programme. However, we lack information on compliance of the implementation of the R4U and Shared Care in daily practice.

A fair question to ask is whether the DiD evaluation of the programme “Ready for a Baby” sufficiently reflects the underlying improvements. The programme had several interacting components of universal and high-risk prevention embedded in improvements in quality of child health care and can therefore be characterized as a complex intervention. Complex interventions usually develop in phases from series of pilots to fully scaled up programs and should preferably be tested using a phased approach, starting with a series of pilot studies and moving on to an exploratory and then a definitive evaluation (27). The programme “Ready for a Baby” could be described as the first phase in the development of a programme to reduce inequalities in perinatal outcomes and the lessons learnt from the programme were included in the next phase, the “Healthy Pregnancy 4 All” programme (28). Therefore, the results of the evaluation of the first programme “Ready for a Baby” should be interpreted with care. Other studies and other research methods are needed to better understand the underlying mechanisms of reach, uptake and effectiveness of specific programme activities (e.g. action research to understand the dynamics of the developing pilots).

In conclusion, in this DiD analysis we could not demonstrate an influence of the urban perinatal health “Ready for a baby” on perinatal outcomes. Epidemiological evidence of inequalities in adverse pregnancy outcomes is compelling enough to justify continued efforts to develop a health care system that can properly deal with social risk factors. It is advised to evaluate such a system when it has been brought to scale and matured

For peer review only - http://bmjopen.bmj.com/site/about/guidelines.xhtml

sufficiently to have a discernible impact (29). The “Ready for a Baby” programme generated a lot of attention both locally and nationally for the relevance of social determinants of pregnancy outcomes and for the development of methods to integrate obstetric care and the social domain, which is a valuable outcome in itself.

Acknowledgements

We'd like to acknowledge Statistics the Netherlands and the Netherlands Perinatal Registry for their support with the data and Semiha Denktas for critically reviewing the manuscript. This research project was conducted as part of the academic collaborative "Centre for Effective Public Health In the larger Rotterdam area" (CEPHIR).

Author contributions

EJ, JB and AB were involved in the conception and design of the study. EJ and JB acquired the data from the Netherlands Perinatal Registry and Statistics the Netherlands. EJ and US analysed the data and all authors were involved with the interpretation of the results. EJ and JL drafted the original work and all authors were involved in revising it critically. All authors have approved the final version of the manuscript.

Competing interests statement

The authors declare no competing interests.

Funding

This research received no specific grant from any funding agency in the public, commercial or not-for-profit sectors'.

For peer review only

Literature

1. project E-p. First european health report. http://wwweuroperistatcom/images/doc/EPHR/european-perinatal-health-reportpdf. Perinatal Health Report. 2008.

2. Mohangoo AD, Buitendijk SE, Hukkelhoven CW, Ravelli AC, Rijninks-van Driel GC, Tamminga P, et al. Higher perinatal mortality in The Netherlands than in other European countries: the Peristat-II study. Ned Tijdschr Geneeskd. 2008;152(50):2718-27.

3. project E-p. The Health and Care of Pregnant Women and Babies in Europe in 2010. Second European Perinatal Health Report. http://wwweuroperistatcom/images/doc/EPHR2010_w_disclaimerpdf. Perinatal healt report. 2013.

4. de Graaf JP, Ravelli AC, Wildschut HI, Denktaş S, Voorham AJ, Bonsel GJ, et al. Perinatal outcomes in the four largest cities and in deprived neighbourhoods in The Netherlands Ned Tijdschr Geneeskd. 2008 Dec 13;152(50):2734-40.

5. Poeran J, Denktas S, Birnie E, Bonsel GJ, Steegers EA. Urban perinatal health inequalities. J Matern Fetal Neonatal Med. 2011;24(4):643-6.

6. Timmermans S, Bonsel GJ, Steegers-Theunissen RP, Mackenbach JP, Steyerberg EW, Raat H, et al. Individual accumulation of heterogeneous risks explains perinatal inequalities within deprived neighbourhoods. Eur J Epidemiol. 2011 Feb;26(2):165-80.

7. Denktas S, Bonsel GJ, Van der Weg EJ, Voorham AJ, Torij HW, De Graaf JP, et al. An urban perinatal health programme of strategies to improve perinatal health. Matern Child Health J. 2012;16(8):1553-8.

8. van der Pal-de Bruin KM, Mohangoo AD, Achterberg PW, Buitendijk SE. Trends in determinanten van perinatale sterfte in Nederland. TSG (2012) 90: 555.

9. Achterberg PW, Waelput AJ. Recente perinatale sterftetrends in Nederland: 2000-2005. Zicht op verbetering. Bilthoven: Rijksinstituut voor Volksgezondheid en Milieu (RIVM); 2007.

10. Craig P, Cooper C, Gunnell D, Haw S, Lawson K, Macintyre S, et al. Using natural experiments to evaluate population health interventions: new Medical Research Council guidance. J Epidemiol Community Health. 2012;66(12):1182-6.

11. Hu Y, van Lenthe FJ, Hoffmann R, van Hedel K, Mackenbach JP. Assessing the impact of natural policy experiments on socioeconomic inequalities in health: how to apply commonly used quantitative analytical methods? BMC Med Res Methodol. 2017 Apr 20;17(1):68.

12. Statistics Netherlands. Urban area definition. https://www.cbs.nl/en-gb/our-services/methods/definitions?tab=u#id=urban-area. Accessed at Jan 11, 2019.

For peer review only - http://bmjopen.bmj.com/site/about/guidelines.xhtml

13. Temel S, Erdem Ö, Voorham TA, Bonsel GJ, Steegers EA, Denktaş S. Knowledge on preconceptional folic acid supplementation and intention to seek for preconception care among men and women in an urban city: a population-based cross-sectional study. BMC Pregnancy Childbirth. 2015; 15: 340.

14. Temel S, Birnie E, Sonneveld HM, Voorham AJ, Bonsel GJ, Steegers EA, Denktaş S. Determinants of the intention of preconception care use: lessons from a multi-ethnic urban population in the Netherlands. Int J Public Health. 2013 Apr;58(2):295-304.

15. Posthumus AG, Schölmerich VL, Waelput AJ, Vos AA, De Jong-Potjer LC, Bakker R, et al. Bridging between professionals in perinatal care: towards shared care in the Netherlands. Matern Child Health J. 2013 Dec;17(10):1981-9.

16. Posthumus AG, Birnie E, van Veen MJ, Steegers EA, Bonsel GJ. An antenatal prediction model for adverse birth outcomes in an urban population: The contribution of medical and non-medical risks. Midwifery. 2016 Jul;38:78-86.

17. van Veen MJ, Birnie E, Poeran J, Torij HW, Steegers EA, Bonsel GJ. Feasibility and reliability of a newly developed antenatal risk score card in routine care. Midwifery. 2015 Jan;31(1):147-54.

18. van der Kooy J, de Graaf JP, Birnie DE, Denktas S, Steegers EA, Bonsel GJ. Different settings of place of midwife-led birth: evaluation of a midwife-led birth centre. Springerplus. 2016 Jun 18;5(1):786.

19. Stehouwer J, van der Kooy J, de Graaf JP, Eggink AJ, Bertens LC, Been JV. Evaluation of the midwife-led primary care Sophia Birth Centre. Ned Tijdschr Geneeskd. [accepted for publication]

20. Jaarboek Zorg 2015. Utrecht: Perined, 2016.

21. Jaarboek Zorg 2009. Utrecht: stichting Perinatale Registratie, 2013.

22. https://www.cbs.nl/-/media/cbs%20op%20maat/microdatabestanden/documents/2016/51/integraal%20persoonlijk%20inkomen.pdf

23. https://www.cbs.nl/-/media/cbs%20op%20maat/microdatabestanden/documents/2017/16/gbaadresobjectbus.pdf

24. Code of Conduct for Medical Research. Council of the Federation of Medical Scientific Societies, 2004.

25. Visser GH, Eilers PH, Elferink-Stinkens PM, Merkus HM, Wit JM. New Dutch reference curves for birthweight by gestational age. Early Hum Dev. 2009 Dec;85(12):737-44.

26. Glymour MM, Avendano M, Kawachi I. Socioeconomic Status and Health. In: Social Epidemiology. Berkman LF, Kawachi I, Glymour MM. Oxford: Oxford University Press; 2014.

27. Craig P, Dieppe P, Macintyre S, Michie S, Nazareth I, Petticrew M. Developing and evaluating complex interventions: the new Medical Research Council guidance. BMJ. 2008 Sep 29;337:a1655.

For peer review only

For peer review only - http://bmjopen.bmj.com/site/about/guidelines.xhtml

28. Denktaş S, Poeran J, van Voorst SF, Vos AA, de Jong-Potjer LC, Waelput AJ, et al. Design and outline of the Healthy Pregnancy 4 All study. BMC Pregnancy Childbirth. 2014 Jul 31;14:253.

29. Waelput AJM, Sijpkens MK, Lagendijk J, van Minde MRC, Raat H, Ernst-Smelt HE, et al. Geographical differences in perinatal health and child welfare in the Netherlands: rationale for the healthy pregnancy 4 all-2 programme. BMC Pregnancy Childbirth. 2017;17(1):254

For peer review only

Figure 1. A graph showing the different parts of the intervention “Ready for a Baby” that were introduced from July 2009 until December 2012.

Figure 2. A flow chart displaying how the study population was generated from the Perined data for the Netherlands, 2003-2014.

For peer review only

For peer review only - http://bmjopen.bmj.com/site/about/guidelines.xhtml

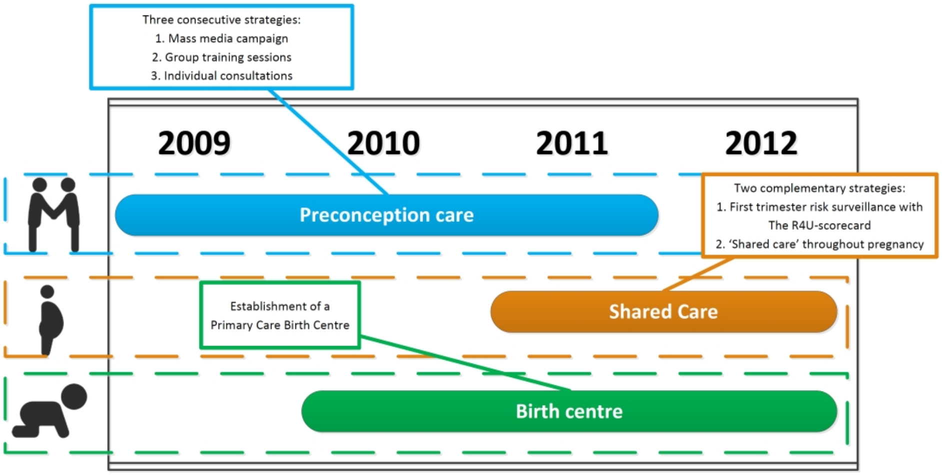

A graph showing the different parts of the intervention "Ready for a Baby" that were introduced from July 2009 until December 2012.

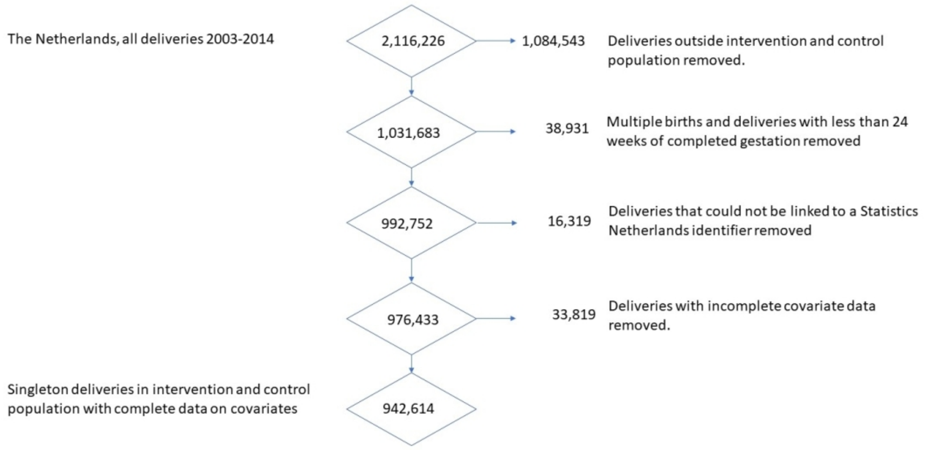

A flow chart displaying how the study population was generated from the Perined data for the Netherlands, 2003-2014.

400x190mm (96 x 96 DPI)

Appendix 1: the number and percentage of low SES pregnant women that are poor by year and the threshold of disposable household income that defines poverty.

Year	No. of deliveries				No. of low SES deliveries				% low SES deliveries			
	Total	NL - other	Control	Intervention	Total	NL - other	Control	Intervention	Total	NL-other	Control	Intervention
2003	187,578	99,171	82,431	5,976	37,749	15,196	19,858	2,695	20%	15%	24%	45%
2004	178,146	93,027	79,504	5,615	35,847	14,253	19,138	2,456	20%	15%	24%	44%
2005	175,231	90,968	78,604	5,659	35,238	13,793	18,983	2,462	20%	15%	24%	44%
2006	174,501	90,425	78,608	5,468	35,080	13,575	19,103	2,401	20%	15%	24%	44%
2007	171,618	88,861	77,465	5,292	34,482	13,275	18,901	2,306	20%	15%	24%	44%
2008	175,762	90,093	80,095	5,574	35,342	13,731	19,335	2,276	20%	15%	24%	41%
2009	178,213	90,717	81,587	5,909	35,789	13,981	19,408	2,401	20%	15%	24%	41%
2010	175,533	88,461	81,501	5,571	35,227	13,623	19,310	2,294	20%	15%	24%	41%
2011	175,346	87,821	81,488	6,037	35,189	13,283	19,357	2,549	20%	15%	24%	42%
2012	172,888	85,136	81,474	6,278	34,695	12,561	19,541	2,593	20%	15%	24%	41%
2013	166,248	81,786	78,553	5,909	33,389	12,331	18,672	2,387	20%	15%	24%	40%
2014	170,837	83,752	81,056	6,029	34,276	12,540	19,372	2,364	20%	15%	24%	39%

For peer review only

The RECORD statement – checklist of items, extended from the STROBE statement, that should be reported in observational studies using routinely collected health data.

	Item No.	STROBE items	Location in manuscript where items are reported	RECORD items	Location in manuscript where items are reported
**Title and abstract**					
	1	(a) Indicate the study's design with a commonly used term in the title or the abstract<br>(b) Provide in the abstract an informative and balanced summary of what was done and what was found	p.1<br><br>p.2	RECORD 1.1: The type of data used should be specified in the title or abstract. When possible, the name of the databases used should be included.<br><br>RECORD 1.2: If applicable, the geographic region and timeframe within which the study took place should be reported in the title or abstract.<br><br>RECORD 1.3: If linkage between databases was conducted for the study, this should be clearly stated in the title or abstract.	p.2 abstract<br><br>p.2 abstract<br><br>p.2 abstract
**Introduction**					
Background rationale	2	Explain the scientific background and rationale for the investigation being reported	p.3		
Objectives	3	State specific objectives, including any prespecified hypotheses	p.3		
**Methods**					
Study Design	4	Present key elements of study design early in the paper	p.4		
Setting	5	Describe the setting, locations, and relevant dates, including periods of recruitment, exposure, follow-up, and data collection	p.4		

For peer review only

Participants	6	*(a) Cohort study* - Give the eligibility criteria, and the sources and methods of selection of participants. Describe methods of follow-up<br>*Case-control study* - Give the eligibility criteria, and the sources and methods of case ascertainment and control selection. Give the rationale for the choice of cases and controls<br>*Cross-sectional study* - Give the eligibility criteria, and the sources and methods of selection of participants<br><br>*(b) Cohort study* - For matched studies, give matching criteria and number of exposed and unexposed<br>*Case-control study* - For matched studies, give matching criteria and the number of controls per case		RECORD 6.1: The methods of study population selection (such as codes or algorithms used to identify subjects) should be listed in detail. If this is not possible, an explanation should be provided.<br><br>RECORD 6.2: Any validation studies of the codes or algorithms used to select the population should be referenced. If validation was conducted for this study and not published elsewhere, detailed methods and results should be provided.<br><br>RECORD 6.3: If the study involved linkage of databases, consider use of a flow diagram or other graphical display to demonstrate the data linkage process, including the number of individuals with linked data at each stage.	6.1 p.5<br><br>**6.2 p.5**<br>Toelichting.<br><br>6.3. figure 2, p.21
Variables	7	Clearly define all outcomes, exposures, predictors, potential confounders, and effect modifiers. Give diagnostic criteria, if applicable.	p.7-8	RECORD 7.1: A complete list of codes and algorithms used to classify exposures, outcomes, confounders, and effect modifiers should be provided. If these cannot be reported, an explanation should be provided.	p.7-8
Data sources/ measurement	8	For each variable of interest, give sources of data and details of methods of assessment (measurement).<br>Describe comparability of assessment methods if there is more than one group	p.7-8		

For peer review only

Bias	9	Describe any efforts to address potential sources of bias	p. 6		
Study size	10	Explain how the study size was arrived at	p. 4, 6		
Quantitative variables	11	Explain how quantitative variables were handled in the analyses. If applicable, describe which groupings were chosen, and why	p. 7		
Statistical methods	12	(a) Describe all statistical methods, including those used to control for confounding<br>(b) Describe any methods used to examine subgroups and interactions<br>(c) Explain how missing data were addressed<br>(d) *Cohort study* - If applicable, explain how loss to follow-up was addressed<br>*Case-control study* - If applicable, explain how matching of cases and controls was addressed<br>*Cross-sectional study* - If applicable, describe analytical methods taking account of sampling strategy<br>(e) Describe any sensitivity analyses	p. 7		
Data access and cleaning methods		..		RECORD 12.1: Authors should describe the extent to which the investigators had access to the database population used to create the study population.	p. 6

For peer review only

				RECORD 12.2: Authors should provide information on the data cleaning methods used in the study.	
Linkage		..		RECORD 12.3: State whether the study included person-level, institutional-level, or other data linkage across two or more databases. The methods of linkage and methods of linkage quality evaluation should be provided.	p. 6
**Results**					
Participants	13	(a) Report the numbers of individuals at each stage of the study (*e.g.*, numbers potentially eligible, examined for eligibility, confirmed eligible, included in the study, completing follow-up, and analysed)<br>(b) Give reasons for non-participation at each stage.<br>(c) Consider use of a flow diagram	p.9	RECORD 13.1: Describe in detail the selection of the persons included in the study (*i.e.,* study population selection) including filtering based on data quality, data availability and linkage. The selection of included persons can be described in the text and/or by means of the study flow diagram.	p. 6 and figure 2. P21
Descriptive data	14	(a) Give characteristics of study participants (*e.g.*, demographic, clinical, social) and information on exposures and potential confounders<br>(b) Indicate the number of participants with missing data for each variable of interest<br>(c) *Cohort study* - summarise follow-up time (*e.g.*, average and total amount)	Table 1<br><br>p. 9		
Outcome data	15	*Cohort study* - Report numbers of outcome events or summary measures over time<br>*Case-control study* - Report numbers in each exposure	p. 9 and Table 1		

		category, or summary measures of exposure<br>*Cross-sectional study* - Report numbers of outcome events or summary measures			
Main results	16	(a) Give unadjusted estimates and, if applicable, confounder-adjusted estimates and their precision (e.g., 95% confidence interval). Make clear which confounders were adjusted for and why they were included<br>(b) Report category boundaries when continuous variables were categorized<br>(c) If relevant, consider translating estimates of relative risk into absolute risk for a meaningful time period	Table 2		
Other analyses	17	Report other analyses done—e.g., analyses of subgroups and interactions, and sensitivity analyses	Table 3		
**Discussion**					
Key results	18	Summarise key results with reference to study objectives	p.11		
Limitations	19	Discuss limitations of the study, taking into account sources of potential bias or imprecision. Discuss both direction and magnitude of any potential bias	p.11-12	RECORD 19.1: Discuss the implications of using data that were not created or collected to answer the specific research question(s). Include discussion of misclassification bias, unmeasured confounding, missing data, and changing eligibility over time, as they pertain to the study being reported.	p.11-12
Interpretation	20	Give a cautious overall interpretation of results considering objectives,	p.12-13		

		limitations, multiplicity of analyses, results from similar studies, and other relevant evidence			
Generalisability	21	Discuss the generalisability (external validity) of the study results	p.12-13		
**Other Information**					
Funding	22	Give the source of funding and the role of the funders for the present study and, if applicable, for the original study on which the present article is based	Information provided to journal		
Accessibility of protocol, raw data, and programming code		..	Information provided to journal	RECORD 22.1: Authors should provide information on how to access any supplemental information such as the study protocol, raw data, or programming code.	Information provided to journal

*Reference: Benchimol EI, Smeeth L, Guttmann A, Harron K, Moher D, Petersen I, Sørensen HT, von Elm E, Langan SM, the RECORD Working Committee. The REporting of studies Conducted using Observational Routinely-collected health Data (RECORD) Statement. *PLoS Medicine* 2015; in press.

*Checklist is protected under Creative Commons Attribution (CC BY) license.

For peer review only

BMJ Open

Did an urban perinatal health programme in Rotterdam, the Netherlands, reduce adverse perinatal outcomes? A register-based retrospective cohort study.

Journal:	*BMJ Open*
Manuscript ID	bmjopen-2019-031357.R1
Article Type:	Original research
Date Submitted by the Author:	18-Sep-2019
Complete List of Authors:	de Jonge, Hendrik C. C.; Erasmus MC, Public Health<br>Lagendijk, Jacqueline; Erasmus MC, Department of Obstetrics and Gynaecology<br>Saha, Unnati; Erasmus MC, Public Health<br>Been, JV; Erasmus University Medical Centre–Sophia Children's Hospital, Paediatrics<br>Burdorf, Alex; Erasmus MC, Department of Public Health
**Primary Subject Heading**:	Public health
Secondary Subject Heading:	Epidemiology, Obstetrics and gynaecology
Keywords:	PUBLIC HEALTH, EPIDEMIOLOGY, PERINATOLOGY

SCHOLARONE™
Manuscripts

Did an urban perinatal health programme in Rotterdam, the Netherlands, reduce adverse perinatal outcomes? A register-based retrospective cohort study.

Hendrik C.C. de Jonge (h.c.c.dejonge@erasmusmc.nl) [1*]

Jacqueline Lagendijk (j.lagendijk.2@erasmusmc.nl) [2]

Unnati Rani Saha (u.saha@erasmusmc.nl) [1]

Jasper V. Been (j.been@erasmusmc.nl) [1,2,3]

Alex Burdorf (a.burdorf@erasmusmc.nl) [1]

Affiliations:

1. Erasmus MC, University Medical Center Rotterdam, Department of Public Health

2. Erasmus MC, University Medical Center Rotterdam, Department of Obstetrics and Gynaecology

3. Erasmus MC - Sophia Children's Hospital, University Medical Center Rotterdam, Department of Pediatrics, Division of(Neonatology

Corresponding author:

Erik de Jonge, Department of Public Health, Erasmus MC, P.O. Box 2040, 3000 CA Rotterdam, the Netherlands.
Email: h.c.c.dejonge@erasmusmc.nl

Number of words (excl. title page, abstract and literature): 4,348 words.

For peer review only

Abstract

Objectives: to study the effect of an urban perinatal health programme in Rotterdam, the Netherlands, on perinatal outcomes.

Design: A retrospective cohort study with difference-in-differences (DiD) analysis using individual-level perinatal outcome data from the Dutch Perinatal Registry 2003-2014 linked to Central Bureau of Statistics data of migration background and individual disposable household income.

Intervention: The programme consisted of perinatal health promotion, risk selection and risk-guided pregnancy care, and a new primary care child birth centre. The programme was implemented during 2009-2012.

Primary outcome measures: We compared trends in perinatal mortality, preterm delivery, and small for gestational age births between targeted urban neighbourhoods in Rotterdam (n=61,415) and all other urban neighbourhoods in the Netherlands (n=881,202). The effect of the programme was modelled as a change in trend of each perinatal outcome in the treatment group post-intervention compared to the control population from January 2010 onwards. All analyses were adjusted for maternal age, parity, ethnicity, and individual-level low socio-economic status (SES). We also conducted a stratified analysis by SES.

Results: During 2003-2014 downward trends in perinatal mortality (adjusted odds ratio (aOR) 0.9439 per year, 95% confidence interval (CI) 0.9362-0.9517), preterm birth (aOR 0.9970 per year, 95% CI 0.9944-0.9997), and small for gestational age births (aOR 0.9809 per year, 95% CI 0.9787-0.9831) in the entire study population were observed. No demonstrable changes in these trends were found in the intervention group after the programme had started. The stratified analyses by SES showed no changes in trends post-intervention in both strata either.

Conclusions: The programme had no demonstrable effects on perinatal outcomes. The intervention may not have reached a sufficient proportion of the population or has provided too little contrast to the widespread attention for inequalities in pregnancy outcomes occurring simultaneously in the Netherlands.

For peer review only

For peer review only - http://bmjopen.bmj.com/site/about/guidelines.xhtml

Strengths & limitations

- The nationally representative sample of almost a million pregnancies during the period 2003-2014 had a good discriminatory power to estimate the effect of the programme on a rare outcome like perinatal mortality.
- The linkage across three routinely collected data registries provided pregnancy outcomes and socioeconomic data, allowing adjustment for important medical and social determinants in the statistical analysis.
- We applied difference-in-differences analysis, an econometric method, that allows us to determine programme effects on population level.
- All pregnant women in the urban neighbourhoods targeted with the intervention were considered to be exposed to the intervention, whether or not these women were actually reached by activities within the complex intervention, which may have attenuated observed associations.
- The relative good performance of the urban perinatal health care system in Rotterdam before the intervention period and unobserved improvements in urban perinatal health care during the intervention period in the Netherlands may have attenuated the observed intervention effect.

For peer review only

For peer review only - http://bmjopen.bmj.com/site/about/guidelines.xhtml

Introduction

In the 2000s the Netherlands had a relatively high perinatal mortality, ranking third highest among 26 European countries in 2004 and sixth highest in 2010 (1-3). Considerable regional inequalities in perinatal outcomes were reported within the Netherlands (4). In Rotterdam, the second largest city in the Netherlands, perinatal mortality was markedly higher than nationally, most notably in its deprived neighbourhoods (5). In a child-birth cohort study, these neighbourhood differences in various perinatal outcomes could largely be attributed to the increased prevalence of medical, as well as, social risk factors (6).

In response to these findings, the Erasmus MC in collaboration with the Municipal Health Service Rotterdam-Rijnmond initiated an urban perinatal health programme, called “Ready for a Baby”, with the aim to improve perinatal outcomes in Rotterdam. The programme consisted of several intervention components across the pregnancy care chain: preconception health promotion, improved risk selection and risk guided care during pregnancy, and the establishment of a primary care birth centre. These components were gradually introduced in the period 2009-2012, and, depending on the component, reached nearly city wide coverage (preconception health promotion) or only specific neighbourhoods (e.g. primary care birth centre) (7).

Perinatal mortality in the Netherlands has gradually reduced over the past two decades. Favourable trends in risk factors may have contributed, including a reduction in smoking by pregnant women, less multiple births, increased use of ultrasonography at 20 weeks gestation for detection of congenital abnormalities, and improved care for very premature babies (8-9). The introduction of the “Ready for a Baby” programme in Rotterdam was conceptualised as a natural experiment (10). In order to evaluate whether this programme has had an additional impact on the secular trends of decline in unfavourable perinatal outcomes, the Difference-in-Differences (DiD) method was considered the appropriate approach to evaluate the effects of an intervention in an observational study. In this method the change in health in the intervention group before and after the introduction of the intervention (difference) can be distinguished from changes in health over time in both the intervention and control groups (differences) (11). Therefore, the aim of this study was to determine the influence of the urban perinatal health programme “Ready for a Baby” on adverse perinatal health outcomes.

For peer review only - http://bmjopen.bmj.com/site/about/guidelines.xhtml

Methods

Study design and population

We conducted a retrospective cohort study on routinely collected birth data from the Dutch Perinatal registry, enriched with national registers with personal information to evaluate the influence of a community intervention with a difference-in-difference analysis. The start in year 2003 was determined by individual information on socio-economic status (SES) becoming available in national registers. The year 2014 was the last year with complete information available at time of this study. The study population consisted of all singleton deliveries in urban neighbourhoods in the Netherlands during the study period, comprising approximately 45% of all deliveries in the Netherlands. A neighbourhood was defined as a postal code area and an urban neighbourhood as a postal code area with more than 1500 houses per square kilometre (12). The intervention group consisted of all deliveries in all 51 urban neighbourhoods in 10 out of 14 boroughs in Rotterdam, where at some point in time during the intervention period a component of the urban perinatal health programme was implemented. The control group comprised all deliveries in other urban postal code areas in the Netherlands, including six untargeted urban neighbourhoods in Rotterdam.

Programme description

The "Ready for a Baby" programme had three components: health promotion in preconception care, improved risk selection and risk guided care, and establishment of a primary care birth centre in the university medical centre. The content of the programme has been published in detail before and will be described here briefly (7).

The first component of the programme aimed to promote preconception health by three strategies. The first strategy aimed to collectively increase awareness of the importance of preconception health through mass media campaigns (including flyers, posters, editorials, and advertisements in local Dutch and Turkish newspapers, on busses and trams, at offices of health care providers, pharmacists, retailers, and at churches and mosques). Besides increasing awareness, these campaigns aimed to promote favourable attitudes and behaviours for a healthy pregnancy, such as the use of folic acid and the cessation of smoking and alcohol use. This strategy targeted all citizens in Rotterdam and was not confined to the intervention group. The second strategy used peer education to increase preconception-related health literacy and motivation to attend preconception care consultations, especially amongst low SES and migrant groups (13). Peer educators recruited about 2,300 participants during the course of the programme for peer education group sessions through their existing network and community meeting places (e.g. mosques and schools). The sessions were interactive, often in multiple languages, and provided participants information on the influence of lifestyle changes on pregnancy outcomes, and additionally advised on where to obtain individual consultations. The third strategy in targeted neighbourhoods was the provision of individual preconception care consultations by general practitioners (GPs) and community midwives. At least 43 couples attended an individual consultation

For peer review only - http://bmjopen.bmj.com/site/about/guidelines.xhtml

after receiving tailored health promotion from a web-based preconception health assessment "Preconceptiewijzer.nl" (13-14).

The second component of the programme aimed to improve risk selection and risk guided care by use of the Rotterdam Reproductive Risk Reduction (R4U) scorecard along with the Shared-Care model (15-17) in the targeted neighbourhoods. The R4U scorecard is a systematic risk assessment in the first trimester of pregnancy focussing on medical and non-medical risk factors related to adverse pregnancy outcomes, including for example migration background and low household income (16-17). The Shared-Care model is an approach to risk guided care that has three elements: 1) continuity of care (e.g. a case manager is assigned to high risk women who need care from different professionals, care pathways are defined for risks identified in the R4U), 2) patient centeredness (e.g. through fostering of self-management, and efforts to combine appointments to different care providers), and 3) interprofessional collaboration (e.g. through formulating a joint set of aims and ambitions for collaboration including care pathways, training in team work, and interprofessional education) (15). The R4U-scorecard guided the care pathways in the Shared-Care model through templates describing the consecutive steps a professional was advised to take to reduce the potential contribution of identified risk factors for adverse perinatal health outcomes. In order to enhance the efficiency and quality of the local antenatal health care, all details of instruments and templates were discussed during meetings with community midwives, obstetricians, and social workers under guidance of a member of the "Ready for a Baby" programme. Since midwives are usually the first point of contact of a pregnant woman, the vast majority (n=46) of all community midwives in the intervention area were trained to use and integrate the R4U scorecard along with the Shared-Care model into their daily practice. In three selected boroughs the programme supported the use of care pathways in midwifery practices and hospitals with multidisciplinary meetings to follow up on high risk cases.

The third component of the programme was the establishment of a primary care birth centre (PCBC) in the Erasmus University Medical Centre in Rotterdam. The PCBC is a separate facility led by community midwives, where women can deliver at their own discretion if a hospital delivery is not medically indicated but social factors make a home delivery less preferable (18-19). The PCBC aimed to provide risk-directed care by assessing the risk status of each woman at different intervals (e.g. upon arrival at the PCBC, during labour, and postpartum).

Figure 1 shows how the different parts of the intervention were introduced from July 2009 until December 2012. The first component of the programme, preconception health promotion, targeted primarily women in selected neighbourhoods, but by nature of the instrument the mass media approach had a reach in all neighbourhoods in Rotterdam. The second component, the risk selection and shared care, was implemented in the selected neighbourhoods. The third component, the new birth centre, primarily served pregnant women from the community midwife practices around Erasmus MC. So the different components were introduced at different moments in time and in different neighbourhoods.

For peer review only - http://bmjopen.bmj.com/site/about/guidelines.xhtml

Data

We used data from the Dutch Perinatal Registry 2003-2014 (Perined, https://www.perined.nl). The Dutch Perinatal Registry is a registry with information provided by midwives, gynaecologists, paediatricians, and general practitioners and contains demographic characteristics, medical risk factors, obstetric history, and pregnancy and neonatal outcomes (20-21). Through individual-level linkage the Dutch Perinatal Registry was enriched with two nationwide registries from Statistics Netherlands. Household income from the Integral Household Income (IHI) register, based on tax information from 2003 onwards (22), was used to define low socio-economic status by lowest 20% disposable household income. The second, the Dutch population register, provided information on migration background (23).

A trusted third party (an in-house service at Statistics Netherlands) merged these datasets using four-digit postal code, birth date of the mother and birth date of the child after which the identifying variables were removed. The merged one-way coded dataset was made available in a secure research environment at Statistics Netherlands for analysis. Results were rigorously checked for identifiability by Statistics the Netherlands before they were released from the secure research environment for publication. This procedure is in accordance with Dutch legislation and the Dutch Code of Conduct for Medical Research for use of anonymous data for research purposes without an explicit informed consent (24).

Deliveries with less than 24 completed weeks of gestation from the dataset were excluded, because most of these deliveries will end in stillbirth. These stillbirths aren't registered in the population registry according to Dutch law so can't be linked to household income and migration background data, which were required for the statistical analysis. Records with other missing covariates for the statistical analysis were also excluded.

Outcomes

Our primary outcomes were: perinatal mortality, preterm birth, and small for gestational age (SGA) birth. Perinatal mortality was defined as the number of stillbirths from 24 completed weeks of gestation or early neonatal deaths (i.e. death of a liveborn baby within the first seven days of life) per 1,000 singleton births. Preterm birth was defined as birth before 37 completed weeks of gestation per 1,000 singleton livebirths. SGA was defined as any baby that was smaller than the 10th centile, corrected for gestational age in weeks and sex, according to the Visser curve, per 1,000 singleton livebirths (25).

Statistical analysis

A DiD analysis was conducted with logistic regression models for perinatal mortality, preterm birth, and SGA as dichotomous dependent variables.

For peer review only - http://bmjopen.bmj.com/site/about/guidelines.xhtml

Each model has three key independent variables and several confounders. The first variable is the year of birth as continuous variable, which captures the trend over time and is expressed by the odds ratio (OR) for trend per year that represents the yearly increase or decrease in likelihood of perinatal outcome. The second variable is a dummy variable with value 1 for the intervention group and value 0 for the control group. This dummy variable reflects differences in perinatal health between intervention and control groups at baseline. The third variable is an interaction term between years since start of the programme as continuous variable and intervention group status. This term corresponds to the DiD estimate, as it presents differences in perinatal health trends between intervention and control groups post-intervention over and above the underlying temporal trends (15). It is important to note that this DiD estimate presents the change in slope of the trend for the intervention group post-intervention. Thus, we did not model the invention as an immediate effect (step function) that introduces a constant difference between intervention and control group from start of the programme until 2014. The programme was gradually introduced from July 2009 onwards and, given the duration of pregnancy, we assumed that the intervention could take effect first from 2010 onwards. Therefore, a DiD analysis on change in the trend per year in the intervention group after 2010 was considered most appropriate.

A crucial assumption in the DiD analysis is the parallel trend assumption (15), i.e. that pre-intervention trends were similar in intervention and control groups over the period 2003-2009. This assumption was assessed with graphs of the perinatal outcomes per year 2003-2009 (supplementary file 1-3). The assumption was also tested with a regression model on the pre-intervention period 2003-2009 with an interaction between intervention and a dummy variable for year of birth 2003-2009, which indicates whether the baseline difference between intervention and control group changed per year. The graphs and regression model showed that the parallel trend assumption was not violated for any of the perinatal outcomes (supplementary file 4).

We included four major risk factors that were targeted in the intervention (SES, ethnicity, parity and age) as confounders. This step was taken to improve the comparability of the intervention and control groups in the analysis. SES is associated with perinatal outcomes and was differently distributed in the intervention and control groups, therefore lowest 20 percentile household income was used as indicator of SES. As the intervention targeted particularly low-SES women, we also conducted stratified analyses according to SES. Ethnicity is associated with perinatal outcomes and with urbanisation, and hence was included as confounder. Non-Dutch ethnicity was defined as any person who was born in another country than the Netherlands (first generation migration background) or had at least one parent born in another country (second generation migration background). We also included parity and age at delivery as potential confounders.

We conducted several sensitivity analyses for lagged programme effects, adjustment for covariate imbalance and the individual program component effects. Lagged programme effects were studied with a regression model with an interaction of the intervention and a dummy variable for each year in the period 2010-2014. Adjustment for covariate imbalance in our main analysis was handled by ordinary regression analysis, which is appropriate given the number of observations (26). Propensity score matching as alternative approach was conducted as a sensitivity analysis (27). For the propensity score model we used the same set of variables for

For peer review only - http://bmjopen.bmj.com/site/about/guidelines.xhtml

matching as in the main analysis (age, parity, migration background, household income). Matching was done per year of delivery using the nearest neighbour algorithm. We evaluated the balance as sufficient by inspecting a table with the distribution of the outcomes and covariates. As a final sensitivity analysis, we studied a possible intervention effect in the boroughs that were targeted by these interventions, compared to the control population, using the same difference-in-difference model as in the main analysis.

Patient and public involvement

This research was done without patient or public involvement. However, the research question was proposed by the Municipal Health Service Rotterdam as part of their legal task of monitoring population health (Dutch Public Health Law).

Results

Study population

From 2003-2014, a total of 2,116,226 deliveries was available in the Dutch Perinatal Registry, encompassing 96.6% of all deliveries in the Netherlands in that period [20-21]. A total of 1,031,683 deliveries was registered within an urban neighbourhood of the intervention and control group, thus representing 49% of all deliveries in the Netherlands (figure 2). We excluded all deliveries before 24 weeks of completed gestation and all multiple births, which were 38,931 deliveries (3.8% of all deliveries) of which 3,704 perinatal deaths (39% of all perinatal deaths (Supplementary File 5)). In addition, we excluded deliveries that could not be matched to Statistics Netherlands data (household income or migration background)(16,319 deliveries, 1.6%) or lacked information on any other confounder (33,819 deliveries, 3.3%). Thus, the total study population comprised 942,614 deliveries (figure 2).

In the intervention group 83% of all deliveries took place in deprived urban neighbourhoods, whereas in the control group the corresponding figure was 39% (table 1). Women delivering in the intervention group were more often younger, multiparous, non-Dutch, and more often had a household income below the 20th percentile. The percentage of women with more than one of these risk factor was considerably higher in the intervention population (59%) than in the control population (34%). In addition, in deprived neighbourhoods in the intervention group pregnant women had a higher prevalence of risk factors than pregnant women in deprived neighbourhoods of the control group, specifically non-Dutch background (73% vs. 49%) and low dispensable household income (46% vs. 35%). The incidence rates for perinatal mortality, preterm birth and for SGA were higher in the intervention group than in the control group, both in deprived as well as non-deprived neighbourhoods.

For peer review only - http://bmjopen.bmj.com/site/about/guidelines.xhtml

Table 1: Socio-demographic characteristics, presence of risk factors, and perinatal mortality, preterm birth, and small-for-gestational age over the period 2003-2014 in intervention group (targeted urban neighbourhoods in Rotterdam) and control group (other urban neighbourhoods in the Netherlands), stratified by deprivation of postal code area.*

	Intervention area						Control areas					
	All		*Deprived*		*Non-deprived*		*All*		*Deprived*		*Non-deprived*	
Total no. of deliveries		61,415		50,727		10,688		881,202		340,914		540,288
Parity												
Nulliparous	48%	29,308	46%	23,180	57%	6,128	47%	418,074	47%	160,270	48%	257,804
1-2	44%	26,880	45%	22,716	39%	4,164	47%	417,538	46%	156,892	48%	260,646
3+	9%	5,227	10%	4,831	4%	396	5%	45,590	7%	23,752	4%	21,838
Ethnicity (non Dutch)	68%	41,702	73%	36,951	44%	4,751	37%	321,950	49%	167,943	29%	154,007
Age of mother												
<25	20%	12,423	22%	11,319	10%	1,104	13%	110,465	18%	60,191	9%	50,274
25-29	30%	18,361	31%	15,679	25%	2,682	28%	250,322	31%	105,834	27%	144,488
30-34	31%	18,917	29%	14,522	41%	4,395	37%	327,361	33%	111,349	40%	216,012
35-39	16%	9,616	15%	7,491	20%	2,125	19%	164,403	16%	53,252	21%	111,151
>=40	3%	2,098	3%	1,716	4%	382	3%	28,651	3%	10,288	3%	18,363
Socio-economic status												
lowest 20% disposable household income	42%	25,919	46%	23,431	23%	2,488	24%	212,405	35%	120,488	17%	91,917
More than 1 risk factor†	59%	36,139	63%	29,638	43%	6,501	34%	299,727	49%	129,418	27%	170,309
Perinatal mortality (/1,000 stillbirths & live singleton births)												

For peer review only - http://bmjopen.bmj.com/site/about/guidelines.xhtml

For peer review only

2003-2009	6.5	223	6.7	202	4.8	21	6.2	3,173	7.1	1,436	5.7	1,737
2010-2014	5.1	136	5.7	116	3.2	20	4.5	1,667	5.2	725	4.0	942
Preterm birth rate (/1,000 live singleton births)												
2003-2009	63	2,166	64	1,917	57	249	56	28447	60	12,066	54	16,381
2010-2014	60	1,611	61	1,254	57	357	55	20376	59	8,098	53	12,278
SGA rate (1,000 live singleton births)												
2003-2009	109	3,756	113	3,377	87	379	84	42,505	96	19,330	76	23,175
2010-2014	94	2,513	98	2,007	80	506	76	28,249	86	11,898	70	16,351

* Deprivation is defined as the lowest quintile of postal codes by status score, an area level composite index of individual household income, education and employment status calculated every four years by the Netherlands Institute of Social Research.

† The following risk factors are considered: nulliparous, non Dutch ethnicity, age of the mother <25 year, lowest 20% disposable household income

For peer review only

For peer review only - http://bmjopen.bmj.com/site/about/guidelines.xhtml

Differences-in-differences analysis

Table 2 shows that the likelihood of perinatal mortality decreased during 2003-2014 by about 6% per year across all urban neighbourhoods in the Netherlands (adjusted odd ratio (aOR) 0.9439/year (95% confidence interval (CI) 0.9362-0.9517)). SGA decreased by 2% per year (aOR 0.9809 (95% CI 0.9787-0.9831)) and preterm birth decreased by 0.3% per year (aOR 0.9970 (95% CI 0.9944-0.9997)). Small differences in annual trends between the intervention and the control group were observed, albeit not statistically significant. The DiD analysis on the effect of the urban perinatal health programme showed that during the post-intervention period in the intervention group perinatal mortality (aOR 1.0535 (95% CI 0.9889-1.1223) increased slightly each year, whereas preterm birth (aOR 0.9809 (95% CI 0.9619-1.0004) and SGA (aOR 0.9928 (95% CI 0.9772-1.0086)) showed modest improvements.

The analysis showed that for the pre-intervention period, after adjustment for important confounders, perinatal mortality was 14% lower in the intervention population than in the control population (aOR 0.8601, 95% CI 0.7534-0.9819). Preterm birth (aOR 1.0962 (95% CI 1.0505-1.1440)) and SGA (aOR 1.1313 (95% CI 1.0935-1.1703)) were higher in the intervention population than in the control population.

For peer review only

For peer review only - http://bmjopen.bmj.com/site/about/guidelines.xhtml

Table 2. Logistic regression models with difference-in-differences analyses of the effect of the urban perinatal health programme on perinatal mortality, preterm birth and SGA.

	Perinatal mortality			Preterm birth			SGA		
independent variables	OR	95% CI		OR	95% CI		OR	95% CI	
trend per year	0.9439	0.9362	0.9517	0.9970	0.9944	0.9997	0.9809	0.9787	0.9831
difference intervention and control group	0.8601	0.7534	0.9819	1.0962	1.0505	1.1440	1.1313	1.0935	1.1703
programme effect (change in trend per year from 2010 onwards)	1.0535	0.9889	1.1223	0.9809	0.9619	1.0004	0.9928	0.9772	1.0086
low SES	1.3761	1.2917	1.4660	1.2017	1.1764	1.2275	1.4417	1.4170	1.4668
ethnicity non Dutch	1.2690	1.3462	1.1963	1.0068	1.0264	0.9876	1.2927	1.3134	1.2722
parity 0 (reference)	1.0000			1.0000			1.0000		
parity 1-2	0.7560	0.7120	0.8026	0.6114	0.5998	0.6232	0.5340	0.5254	0.5427
parity 3+	1.2823	1.1541	1.4248	0.8218	0.7894	0.8554	0.4871	0.4687	0.5062
age <25 (reference)	1.0000			1.0000			1.0000		
age 25-34	0.9401	0.8654	1.0212	0.9786	0.9529	1.0050	0.8554	0.8377	0.8735
age >=35	1.2238	1.1112	1.3479	1.0762	1.0423	1.1111	0.9339	0.9100	0.9585
baseline rate*	8.6	7.8	9.4	71	69	74	155	151	159

Perinatal mortality is defined as still birth from 24 weeks onwards plus early neonatal mortality. The total number of observations for the perinatal mortality model is 942,614. Preterm is defined as born before a gestational age of 37 weeks and SGA is defined as a birth weight below the 10th percentile corrected for gestational age and sex. The total number of observations for the preterm birth and SGA models is 937,639.

* for perinatal mortality per 1,000 live and stillbirths, for preterm birth and SGA per 1,000 live births

For peer review only

The stratified analysis by socio-economic status of the pregnant woman showed very similar results for pregnant women with low SES and pregnant women with higher SES. The intervention had no demonstrable influence on trends in any of the perinatal outcomes post-intervention (table 3).

The sensitivity analysis for lagged programme effects did not indicate any lagged programme effect (supplementary file 6). The sensitivity analysis using propensity score matching gave similar results to the main analysis (supplementary file 7). No intervention effects were observed for individual programme components (supplementary file 8).

For peer review only

Table 3. Stratified analysis by socio-economic status of the mother with logistic regression models with difference-in-differences analyses of the effect of the urban perinatal health programme on perinatal mortality, preterm birth and SGA.

**Lowest quintile household income**	Perinatal mortality			Preterm birth			SGA		
independent variables	OR	95% CI		OR	95% CI		OR	95% CI	
trend per year	0.9516	0.9380	0.9654	1.0008	0.9958	1.0059	0.9864	0.9825	0.9903
difference intervention and control group	0.8209	0.6786	0.9929	1.1677	1.0957	1.2444	1.1296	1.0749	1.1871
programme effect (change in trend per year from 2010 onwards)	1.0673	0.9761	1.1670	0.9767	0.9481	1.0062	0.9947	0.9718	1.0181
ethnicity non Dutch	1.3668	1.5181	1.2307	0.8716	0.9024	0.8418	1.0167	1.0450	0.9893
parity 0 (reference)	1.0000			1.0000			1.0000		
parity 1-2	0.7392	0.6652	0.8214	0.7826	0.7545	0.8117	0.6258	0.6084	0.6438
parity 3	1.0544	0.8960	1.2407	1.0134	0.9539	1.0766	0.5605	0.5308	0.5918
age <25 (reference)	1.0000			1.0000			1.0000		
age 25-34	1.0666	0.9478	1.2004	0.9566	0.9188	0.9960	0.8914	0.8647	0.9190
age >=35	1.2893	1.1082	1.4999	1.1172	1.0597	1.1778	0.9334	0.8947	0.9737
baseline rate*	11.2	9.8	12.7	70	67	74	181	175	188

**Household income above 20th percentile**	Perinatal mortality			Preterm birth			SGA		
independent variables	OR	95% CI		OR	95% CI		OR	95% CI	
trend per year	0.9402	0.9308	0.9497	0.9961	0.9930	0.9992	0.9787	0.9761	0.9813
difference intervention and control group	0.9084	0.7553	1.0926	1.0472	0.9884	1.1094	1.1500	1.0978	1.2046
intervention effect (change in trend per year from 2010 onwards)	1.0294	0.9403	1.1270	0.9841	0.9585	1.0103	0.9876	0.9663	1.0093
ethnicity non Dutch	1.2108	1.3017	1.1262	1.0665	1.0913	1.0423	1.4399	1.4678	1.4126
parity 0 (reference)	1.0000			1.0000			1.0000		
parity 1-2	0.7592	0.7057	0.8166	0.5592	0.5467	0.5721	0.4956	0.4859	0.5056

For peer review only - http://bmjopen.bmj.com/site/about/guidelines.xhtml

For peer review only

parity 3+	1.4987	1.3058	1.7201	0.7298	0.6905	0.7713	0.4446	0.4206	0.4700
age <25 (reference)	1.0000			1.0000			1.0000		
age 25-34	0.8417	0.7489	0.9461	0.9544	0.9206	0.9894	0.8185	0.7950	0.8427
age >=35	1.1253	0.9880	1.2816	1.0446	1.0024	1.0886	0.9142	0.8835	0.9459
baseline rate*	9.2	8.1	10.4	80	76	83	179	173	185

Perinatal mortality is defined as still birth from 24 weeks onwards plus early neonatal mortality. The total number of observations for the perinatal mortality model is 238,226 for low SES and 704,388 for high SES. Preterm is defined as born before a gestational age of 37 weeks and SGA is defined as a birth weight below the 10th percentile corrected for gestational age and sex. The total number of observations for the preterm birth and SGA models is 236,737 for low SES and 701,524 for high SES. Low SES was defined as the lowest 20 percentile by disposable household income per year.

* for perinatal mortality per 1,000 live and stillbirths, for preterm birth and SGA per 1,000 live births

For peer review only - http://bmjopen.bmj.com/site/about/guidelines.xhtml

For peer review only

Discussion

In this study, the influence of an urban perinatal health programme in Rotterdam on perinatal health outcomes was evaluated by a DiD approach, an analytical technique for natural experiment evaluation in an observational setting. The DiD analysis could not demonstrate that the introduction of the programme influenced trends in perinatal mortality, preterm birth, or small-for-gestational age birth in the post-intervention years in the intervention group.

Strengths of this study include the large study population and the available information on important confounders. The nationally representative sample of almost a million pregnancies during the period 2003-2014 had a good discriminatory power to estimate the effect of the programme on a rare outcome like perinatal mortality. The linkage across three routinely collected data registries provided pregnancy outcomes and socioeconomic data, allowing adjustment for important medical and social determinants in the statistical analysis. The modelling was checked for robustness by several sensitivity analyses.

Our study also has several limitations pertaining to data availability, and to the DiD analysis. Availability of data was dictated by the registers used. SES is an important determinant of perinatal health (28), but in our analysis only disposable household income was available. Highest educational attainment based on certified diploma registers had a high percentage of missing values, and could therefore not be included as a covariate in our study. Using only a one-dimensional representation of SES might not fully adjust for residual differences between the intervention and the control group.

The DiD analysis applied in this study has several limitations. First, a crucial assumption is that trends in outcome in intervention and control groups in the years before the intervention are parallel, i.e. have a constant difference, captured in the DiD logistic regression model by the difference at baseline (table 2). Evaluation showed that this assumption was not violated. The DiD analysis accounts for time-invariant differences between the intervention and control groups, as well as any factors that equally change over time in both groups. The descriptive information showed that in the intervention neighbourhoods in Rotterdam there is much higher accumulation of risk factors among pregnant women, and that a much larger proportion of women lived in deprived neighbourhoods. In our analysis we adjusted for these risk factors to ensure comparability of intervention and control groups. However, it cannot be discarded that women in Rotterdam have experienced less favourable trends in other, unobserved, risk factors during the observation period of this study, which may have attenuated any potential effect of the intervention. Second, the DiD makes the strong assumption that the precise timing of the intervention is completely at random, creating exogenous variation that allows causal inference. This assumption cannot be formally tested, but it must be acknowledged that the urban perinatal health programme was designed and implemented in response to the relatively high perinatal mortality in the Netherlands and opportunities for improvement in prevention and child health care in Rotterdam.

At the start of the "Ready for a Baby" programme, adjusted for risk factors, perinatal mortality was lower in Rotterdam than in other urban areas in the Netherlands. This favourable position of Rotterdam might be partly

For peer review only - http://bmjopen.bmj.com/site/about/guidelines.xhtml

attributed to the large concentration of hospitals in Rotterdam and attention in the local child health care system for high-risk women since they constitute a relatively large part of all pregnant women. Therefore, the DiD analysis may have not been able to capture additional change in an already decreasing trend in perinatal mortality in the intervention group. A linked issue is that improvements in perinatal health care may have occurred also in other neighbourhoods. These co-interventions may have biased the comparisons between intervention and control groups.

It is important to consider that this study evaluates the possible influence of a population intervention rather than the effects of an intervention at individual level. The content of the complex intervention comprises universal primary prevention and changes in quality and delivery of child health care, which are notoriously difficult to evaluate at individual level. Also, the different components of the intervention were introduced gradually during the intervention period and had varying coverage: from nearly city wide to certain neighbourhoods only. In the analysis, all pregnant women in the targeted urban neighbourhoods with the intervention were considered to be exposed to the intervention, whether or not these women were actually reached by activities within the complex intervention. This might have attenuated observed associations, when a substantial number of women would not have been included in components of the programme. Only 43 couples attended an individual preconception consultation by a general practitioner in all of Rotterdam, which is too small to expect any effect on population level. In contrast, the majority of midwifes was trained to use the R4U scorecard and Shared Care method, resulting in a large uptake of the second component of the programme. However, we lack information on compliance of the implementation of the R4U and Shared Care in daily practice.

A fair question to ask is whether the DiD evaluation of the programme “Ready for a Baby” sufficiently reflects the underlying improvements. The programme had several interacting components of universal and high-risk prevention embedded in improvements in quality of child health care and can therefore be characterized as a complex intervention. Complex interventions usually develop in phases from series of pilots to fully scaled up programs and should preferably be tested using a phased approach, starting with a series of pilot studies and moving on to an exploratory and then a definitive evaluation (29). The programme “Ready for a Baby” could be described as the first phase in the development of a programme to reduce inequalities in perinatal outcomes and the lessons learnt from the programme were included in the next phase, the “Healthy Pregnancy 4 All” programme (30). Therefore, the results of the evaluation of the first programme “Ready for a Baby” should be interpreted with care. Other studies and other research methods are needed to better understand the underlying mechanisms of reach, uptake and effectiveness of specific programme activities (e.g. action research to understand the dynamics of the developing pilots).

In conclusion, in this DiD analysis we could not demonstrate an influence of the urban perinatal health “Ready for a baby” on perinatal outcomes. Epidemiological evidence of inequalities in adverse pregnancy outcomes is compelling enough to justify continued efforts to develop a health care system that can properly deal with social risk factors. It is advised to evaluate such a system when it has been brought to scale and matured sufficiently to have a discernible impact (31). The “Ready for a Baby” programme generated a lot of attention

For peer review only - http://bmjopen.bmj.com/site/about/guidelines.xhtml

both locally and nationally for the relevance of social determinants of pregnancy outcomes and for the development of methods to integrate obstetric care and the social domain, which is a valuable outcome in itself.

Acknowledgements

We'd like to acknowledge Statistics the Netherlands and the Netherlands Perinatal Registry for their support with the data. We'd like to thank Yannan Hu and Marti Rado for their methodological support and Semiha Denktas for critically reviewing the manuscript. This research project was conducted as part of the academic collaborative "Centre for Effective Public Health In the larger Rotterdam area" (CEPHIR).

Data sharing statement

Results are based on calculations by Erasmus MC using non-public microdata from Statistics Netherlands. Under certain conditions, these microdata are accessible for statistical and scientific research. For further information: cvb@cbs.nl. Syntax files that allow repeating the analyses in this paper from microdata at Statistics the Netherlands can be obtained from the first author.

Author contributions

EJ, JB and AB were involved in the conception and design of the study. EJ and JB acquired the data from the Netherlands Perinatal Registry and Statistics the Netherlands. EJ and US analysed the data and all authors were involved with the interpretation of the results. EJ and JL drafted the original work and all authors were involved in revising it critically. All authors have approved the final version of the manuscript.

Competing interests statement

The authors declare no competing interests.

Funding

This research received no specific grant from any funding agency in the public, commercial or not-for-profit sectors'. JVB is supported by a personal fellowship from the Netherlands Lung Foundation.

For peer review only

Literature

1. project E-p. First european health report. http://wwweuroperistatcom/images/doc/EPHR/european-perinatal-health-reportpdf. Perinatal Health Report. 2008.

2. Mohangoo AD, Buitendijk SE, Hukkelhoven CW, Ravelli AC, Rijninks-van Driel GC, Tamminga P, et al. Higher perinatal mortality in The Netherlands than in other European countries: the Peristat-II study. Ned Tijdschr Geneeskd. 2008;152(50):2718-27.

3. project E-p. The Health and Care of Pregnant Women and Babies in Europe in 2010. Second European Perinatal Health Report. http://wwweuroperistatcom/images/doc/EPHR2010_w_disclaimerpdf. Perinatal healt report. 2013.

4. de Graaf JP, Ravelli AC, Wildschut HI, Denktaş S, Voorham AJ, Bonsel GJ, et al. Perinatal outcomes in the four largest cities and in deprived neighbourhoods in The Netherlands Ned Tijdschr Geneeskd. 2008 Dec 13;152(50):2734-40.

5. Poeran J, Denktas S, Birnie E, Bonsel GJ, Steegers EA. Urban perinatal health inequalities. J Matern Fetal Neonatal Med. 2011;24(4):643-6.

6. Timmermans S, Bonsel GJ, Steegers-Theunissen RP, Mackenbach JP, Steyerberg EW, Raat H, et al. Individual accumulation of heterogeneous risks explains perinatal inequalities within deprived neighbourhoods. Eur J Epidemiol. 2011 Feb;26(2):165-80.

7. Denktas S, Bonsel GJ, Van der Weg EJ, Voorham AJ, Torij HW, De Graaf JP, et al. An urban perinatal health programme of strategies to improve perinatal health. Matern Child Health J. 2012;16(8):1553-8.

8. van der Pal-de Bruin KM, Mohangoo AD, Achterberg PW, Buitendijk SE. Trends in determinanten van perinatale sterfte in Nederland. TSG (2012) 90: 555.

9. Achterberg PW, Waelput AJ. Recente perinatale sterftetrends in Nederland: 2000-2005. Zicht op verbetering. Bilthoven: Rijksinstituut voor Volksgezondheid en Milieu (RIVM); 2007.

10. Craig P, Cooper C, Gunnell D, Haw S, Lawson K, Macintyre S, et al. Using natural experiments to evaluate population health interventions: new Medical Research Council guidance. J Epidemiol Community Health. 2012;66(12):1182-6.

11. Hu Y, van Lenthe FJ, Hoffmann R, van Hedel K, Mackenbach JP. Assessing the impact of natural policy experiments on socioeconomic inequalities in health: how to apply commonly used quantitative analytical methods? BMC Med Res Methodol. 2017 Apr 20;17(1):68.

12. Statistics Netherlands. Urban area definition. https://www.cbs.nl/en-gb/our-services/methods/definitions?tab=u#id=urban-area. Accessed at Jan 11, 2019.

For peer review only - http://bmjopen.bmj.com/site/about/guidelines.xhtml

13. Temel S, Erdem Ö, Voorham TA, Bonsel GJ, Steegers EA, Denktaş S. Knowledge on preconceptional folic acid supplementation and intention to seek for preconception care among men and women in an urban city: a population-based cross-sectional study. BMC Pregnancy Childbirth. 2015; 15: 340.

14. Temel S, Birnie E, Sonneveld HM, Voorham AJ, Bonsel GJ, Steegers EA, Denktaş S. Determinants of the intention of preconception care use: lessons from a multi-ethnic urban population in the Netherlands. Int J Public Health. 2013 Apr;58(2):295-304.

15. Posthumus AG, Schölmerich VL, Waelput AJ, Vos AA, De Jong-Potjer LC, Bakker R, et al. Bridging between professionals in perinatal care: towards shared care in the Netherlands. Matern Child Health J. 2013 Dec;17(10):1981-9.

16. Posthumus AG, Birnie E, van Veen MJ, Steegers EA, Bonsel GJ. An antenatal prediction model for adverse birth outcomes in an urban population: The contribution of medical and non-medical risks. Midwifery. 2016 Jul;38:78-86.

17. van Veen MJ, Birnie E, Poeran J, Torij HW, Steegers EA, Bonsel GJ. Feasibility and reliability of a newly developed antenatal risk score card in routine care. Midwifery. 2015 Jan;31(1):147-54.

18. van der Kooy J, de Graaf JP, Birnie DE, Denktas S, Steegers EA, Bonsel GJ. Different settings of place of midwife-led birth: evaluation of a midwife-led birth centre. Springerplus. 2016 Jun 18;5(1):786.

19. Stehouwer J, van der Kooy J, de Graaf JP, Eggink AJ, Bertens LC, Been JV. Evaluation of the midwife-led primary care Sophia Birth Centre. Ned Tijdschr Geneeskd. [accepted for publication]

20. Jaarboek Zorg 2015. Utrecht: Perined, 2016.

21. Jaarboek Zorg 2009. Utrecht: stichting Perinatale Registratie, 2013.

22. Documentatierapport Integraal Persoonlijk Inkomen (IPI). Zoetermeer: Statistics the Netherlands, 2016.

23. Documentatierapport Adreskenmerken van personen die in de gemeentelijke bevolkingsregisters ingeschreven (hebben ge)staan. Zoetermeer: Statistics the Netherlands, 2017.

24. Code of Conduct for Medical Research. Council of the Federation of Medical Scientific Societies, 2004.

25. Visser GH, Eilers PH, Elferink-Stinkens PM, Merkus HM, Wit JM. New Dutch reference curves for birthweight by gestational age. Early Hum Dev. 2009 Dec;85(12):737-44.

26. Soledad Cepeda M, Boston R, Farrar JT, Strom BL. Comparison of Logistic Regression versus Propensity Score When the Number of Events Is Low and There Are Multiple Confounders . American Journal of Epidemiology, 2003; 158(3):280–287.

27. Stuart EA. Matching Methods for Causal Inference: A Review and a Look Forward. Statist. Sci. Volume 25, Number 1 (2010), 1-21.

For peer review only - http://bmjopen.bmj.com/site/about/guidelines.xhtml

28. Glymour MM, Avendano M, Kawachi I. Socioeconomic Status and Health. In: Social Epidemiology. Berkman LF, Kawachi I, Glymour MM. Oxford: Oxford University Press; 2014.

29. Craig P, Dieppe P, Macintyre S, Michie S, Nazareth I, Petticrew M. Developing and evaluating complex interventions: the new Medical Research Council guidance. BMJ. 2008 Sep 29;337:a1655.

30. Denktaş S, Poeran J, van Voorst SF, Vos AA, de Jong-Potjer LC, Waelput AJ, et al. Design and outline of the Healthy Pregnancy 4 All study. BMC Pregnancy Childbirth. 2014 Jul 31;14:253.

31. Waelput AJM, Sijpkens MK, Lagendijk J, van Minde MRC, Raat H, Ernst-Smelt HE, et al. Geographical differences in perinatal health and child welfare in the Netherlands: rationale for the healthy pregnancy 4 all-2 programme. BMC Pregnancy Childbirth. 2017;17(1):254

For peer review only

For peer review only - http://bmjopen.bmj.com/site/about/guidelines.xhtml

Figure 1. A graph showing the different parts of the intervention “Ready for a Baby” that were introduced from July 2009 until December 2012.

Figure 2. A flow chart displaying how the study population was generated from the Perined data for the Netherlands, 2003-2014.

For peer review only

For peer review only - http://bmjopen.bmj.com/site/about/guidelines.xhtml

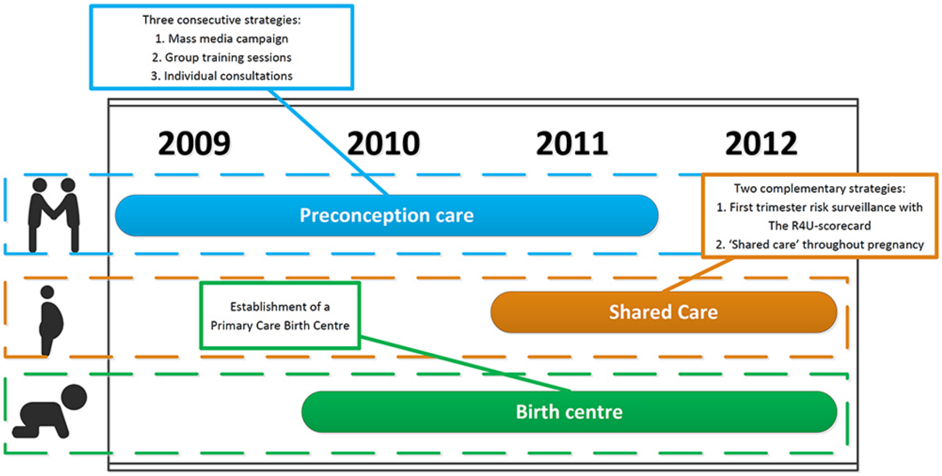

A flow chart displaying how the study population was generated from the Perined data for the Netherlands, 2003-2014.

90x90mm (300 x 300 DPI)

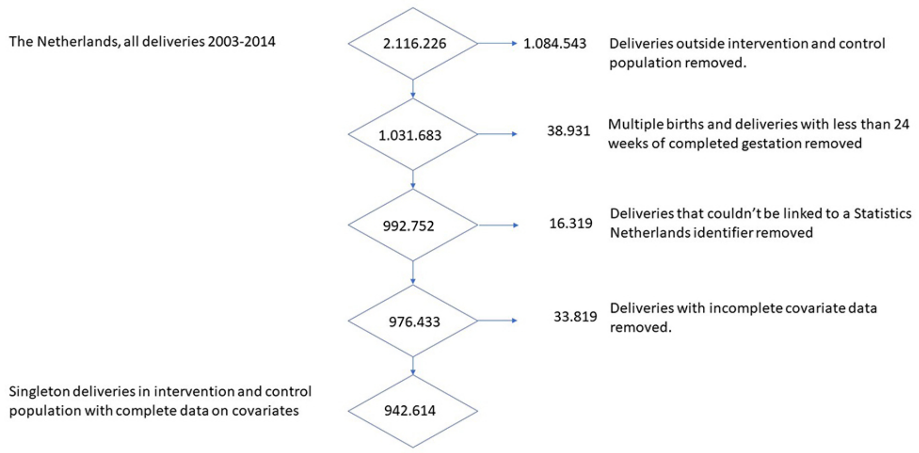

A graph showing the different parts of the intervention "Ready for a Baby" that were introduced from July 2009 until December 2012.

90x90mm (300 x 300 DPI)

Supplementary file 1. Perinatal mortality by year for the control (blue line) and intervention area (red line). Perinatal mortality is defined as still birth from 24 weeks onwards plus early neonatal mortality per 1,000 births.

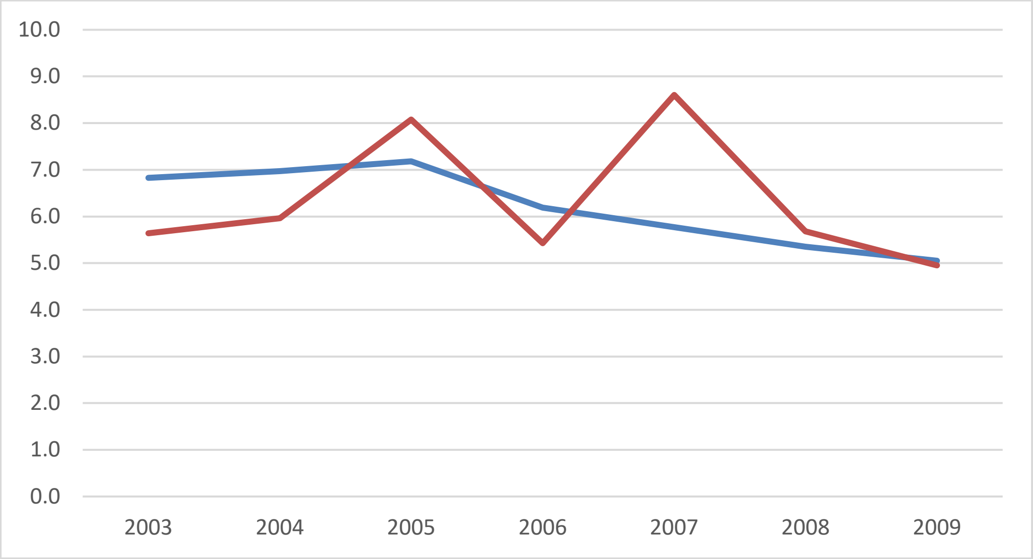

For peer review only - http://bmjopen.bmj.com/site/about/guidelines.xhtml

Supplementary file 2. Preterm birth by year for the control (blue line) and intervention area (orange line). Preterm birth is defined as born before a gestational age of 37 weeks per 1,000 live births.

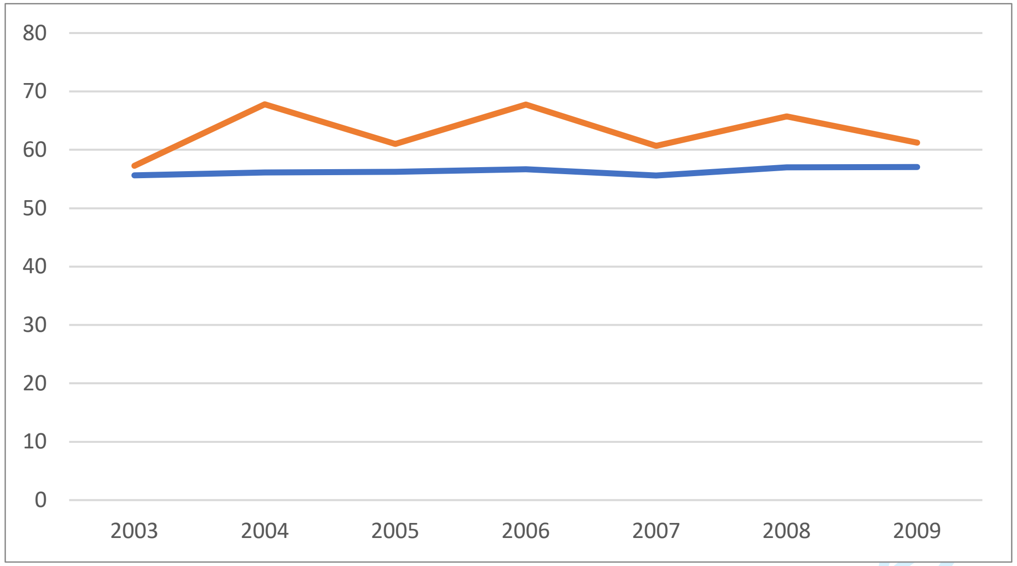

For peer review only - http://bmjopen.bmj.com/site/about/guidelines.xhtml

Supplementary file 3. Small for gestational age (SGA) for the control (blue line) and intervention area (red line). SGA is defined as a birth weight below the 10th percentile for gestational age per 1,000 live births.

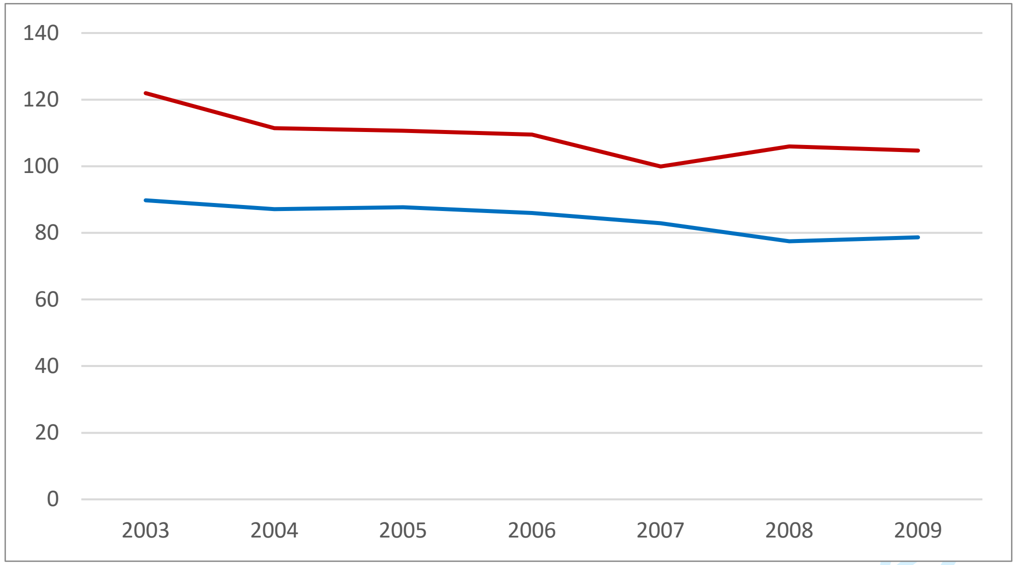

Supplementary file 4. Logistic regression models that test the parallel trend assumption for perinatal mortality, small for gestational age and preterm birth during the preintervention period 2003-2009. Perinatal mortality is defined as still birth from 24 weeks onwards plus early neonatal mortality. Preterm is defined as born before a gestational age of 37 weeks. SGA is defined as a birth weight below the 10th percentile for gestational age. The time trend is defined as a continuous variable per year with 2003 as reference. Possible deviations from the time trend are defined as interactions between time dummies (reference 2003, per year) * intervention. The total number of observations is 542,824 for perinatal mortality and 539,909 for SGA and preterm birth.

	Perinatal mortality			SGA			Preterm birth		
independent variables	OR	95% CI		OR	95% CI		OR	95% CI	
Time (years, 2003-2009, reference 2003, continuous variable)	0.942	0.926	0.959	0.975	0.970	0.979	1.004	0.998	1.010
difference intervention and control	0.654	0.452	0.948	1.184	1.086	1.291	0.976	0.865	1.101
*interaction time (dummy)*intervention (reference 2003)*									
2004	1.127	0.672	1.889	0.933	0.825	1.056	1.195	1.015	1.407
2005	1.620	1.002	2.621	0.958	0.846	1.084	1.069	0.905	1.263
2006	1.158	0.679	1.975	0.980	0.865	1.111	1.203	1.021	1.419
2007	1.938	1.193	3.148	0.910	0.799	1.037	1.060	0.893	1.257
2008	1.376	0.812	2.334	0.999	0.879	1.134	1.149	0.973	1.357
2009	1.272	0.740	2.185	1.018	0.897	1.155	1.064	0.900	1.258
poverty	1.318	1.218	1.425	1.432	1.400	1.464	1.189	1.157	1.223
dutch	0.783	0.728	0.842	0.794	0.778	0.811	0.985	0.960	1.010
parity (n=2)	0.737	0.684	0.794	0.533	0.522	0.544	0.606	0.591	0.621
parity (n=3+)	1.292	1.133	1.473	0.492	0.467	0.517	0.804	0.762	0.848
age 25-34	0.893	0.809	0.987	0.845	0.823	0.868	0.971	0.939	1.005
age >=35	1.179	1.049	1.325	0.912	0.882	0.943	1.055	1.012	1.099

For peer review only

Supplementary file 5: the number and percentage of low SES pregnant women that are poor by year and the threshold of disposable household income that defines poverty.

Year	No. of deliveries				No. of low SES deliveries				% low SES deliveries			
	Total	NL - other	Control	Intervention	Total	NL - other	Control	Intervention	Total	NL-other	Control	Intervention
2003	187,578	99,171	82,431	5,976	37,749	15,196	19,858	2,695	20%	15%	24%	45%
2004	178,146	93,027	79,504	5,615	35,847	14,253	19,138	2,456	20%	15%	24%	44%
2005	175,231	90,968	78,604	5,659	35,238	13,793	18,983	2,462	20%	15%	24%	44%
2006	174,501	90,425	78,608	5,468	35,080	13,575	19,103	2,401	20%	15%	24%	44%
2007	171,618	88,861	77,465	5,292	34,482	13,275	18,901	2,306	20%	15%	24%	44%
2008	175,762	90,093	80,095	5,574	35,342	13,731	19,335	2,276	20%	15%	24%	41%
2009	178,213	90,717	81,587	5,909	35,789	13,981	19,408	2,401	20%	15%	24%	41%
2010	175,533	88,461	81,501	5,571	35,227	13,623	19,310	2,294	20%	15%	24%	41%
2011	175,346	87,821	81,488	6,037	35,189	13,283	19,357	2,549	20%	15%	24%	42%
2012	172,888	85,136	81,474	6,278	34,695	12,561	19,541	2,593	20%	15%	24%	41%
2013	166,248	81,786	78,553	5,909	33,389	12,331	18,672	2,387	20%	15%	24%	40%
2014	170,837	83,752	81,056	6,029	34,276	12,540	19,372	2,364	20%	15%	24%	39%

For peer review only

Supplementary file 6: Lagged treatment effect post intervention (dummies 2010-2014). Perinatal mortality is defined as still birth from 24 weeks onwards plus early neonatal mortality. Preterm is defined as born before a gestational age of 37 weeks. SGA is defined as a birth weight below the 10th percentile for gestational age. The total number of observations is 542,824 for perinatal mortality and 539,909 for SGA and preterm birth.

	Perinatal mortality			SGA			Preterm birth		
independent variables	OR	95% CI		OR	95% CI		OR	95% CI	
difference intervention and control before intervention	0.874	0.761	1.004	1.143	1.103	1.185	1.079	1.031	1.129
change in control area year 1 post intervention (2010)	0.807	0.725	0.897	0.911	0.885	0.938	1.017	0.984	1.051
change in control area year 2 post intervention (2011)	0.716	0.640	0.802	0.898	0.873	0.925	0.973	0.940	1.006
change in control area year 3 post intervention (2012)	0.758	0.679	0.847	0.876	0.851	0.902	0.944	0.912	0.976
change in control area year 4 post intervention (2013)	0.650	0.577	0.733	0.897	0.871	0.924	0.977	0.944	1.011
change in control area year 5 post intervention (2014)	0.608	0.538	0.687	0.891	0.865	0.917	0.957	0.925	0.991
intervention effect year 1 post intervention	1.169	0.786	1.740	0.955	0.860	1.060	1.081	0.956	1.221
intervention effect year 2 post intervention	0.724	0.442	1.188	0.983	0.888	1.087	1.020	0.902	1.153
intervention effect year 3 post intervention	1.074	0.717	1.608	0.925	0.835	1.025	0.932	0.821	1.058
intervention effect year 4 post intervention	1.254	0.825	1.905	0.964	0.870	1.068	0.920	0.809	1.047
intervention effect year 5 post intervention	1.465	0.980	2.189	0.947	0.854	1.050	0.956	0.842	1.086
poverty	1.377	1.292	1.467	1.442	1.417	1.467	1.202	1.176	1.228
dutch	0.790	0.745	0.838	0.774	0.762	0.787	0.993	0.974	1.012
parity (n=2)	0.756	0.712	0.802	0.534	0.525	0.543	0.612	0.600	0.623
parity (n=3+)	1.284	1.156	1.427	0.487	0.469	0.506	0.822	0.790	0.856
age 25-34	0.937	0.862	1.018	0.854	0.836	0.872	0.979	0.953	1.005
age >=35	1.214	1.103	1.337	0.931	0.907	0.956	1.076	1.042	1.111

For peer review only

supplementary file 7: Propensity score matching. Perinatal mortality is defined as still birth from 24 weeks onwards plus early neonatal mortality. Preterm is defined as born before a gestational age of 37 weeks. SGA is defined as a birth weight below the 10th percentile for gestational age. The total number of observations is 122,766 for perinatal mortality and 122,133 for SGA and preterm birth.

	Perinatal mortality			SGA			Preterm birth		
independent variables	OR	95% CI		OR	95% CI		OR	95% CI	
linear time	0.970	0.945	0.995	0.983	0.975	0.990	0.997	0.988	1.006
difference intervention and control	0.920	0.775	1.091	1.254	1.197	1.312	1.207	1.140	1.279
intervention effect - slope change	1.003	0.929	1.084	0.990	0.970	1.010	0.982	0.958	1.006
poverty	1.287	1.103	1.502	1.316	1.263	1.372	1.239	1.176	1.305
dutch	0.654	0.544	0.786	0.718	0.686	0.752	1.020	0.965	1.078
parity (n=2)	0.876	0.744	1.032	0.577	0.553	0.603	0.762	0.722	0.804
parity (n=3+)	1.353	1.056	1.733	0.489	0.449	0.533	0.989	0.902	1.084
age 25-34	0.985	0.811	1.195	0.845	0.805	0.886	0.898	0.844	0.956
age >=35	1.132	0.885	1.448	0.858	0.803	0.917	0.965	0.889	1.047

For peer review only

supplementary file 8: The intervention effect (slope change per year) of three interventions in selected boroughs (peer education, care pathways and birth centre) compared to the control population, using the same difference-in-difference model as in the main analysis. Perinatal mortality is defined as still birth from 24 weeks onwards plus early neonatal mortality. Preterm is defined as born before a gestational age of 37 weeks. SGA is defined as a birth weight below the 10th percentile for gestational age. The analyses are adjusted for time (year), difference between intervention and control group at t=0 (2003), age, parity, migration background and household income.

	perinatal mortality			SGA			preterm birth		
	OR	*95% CI*		*beta*	*95% CI*		*OR*	*95% CI*	
peer education	1.1826	1.0393	1.3457	0.9825	0.9489	1.0171	0.9634	0.9226	1.0059
care pathways	1.1165	0.9512	1.3104	0.9951	0.9552	1.0366	0.9855	0.9364	1.0372
birth centre	1.0583	0.9606	1.1659	0.9896	0.7054	1.0139	0.9706	0.9414	1.0008

For peer review only

The RECORD statement – checklist of items, extended from the STROBE statement, that should be reported in observational studies using routinely collected health data.

	Item No.	STROBE items	Location in manuscript where items are reported	RECORD items	Location in manuscript where items are reported
**Title and abstract**					
	1	(a) Indicate the study's design with a commonly used term in the title or the abstract<br>(b) Provide in the abstract an informative and balanced summary of what was done and what was found	p.1<br><br>p.2	RECORD 1.1: The type of data used should be specified in the title or abstract. When possible, the name of the databases used should be included.<br><br>RECORD 1.2: If applicable, the geographic region and timeframe within which the study took place should be reported in the title or abstract.<br><br>RECORD 1.3: If linkage between databases was conducted for the study, this should be clearly stated in the title or abstract.	p.2 abstract<br><br>p.2 abstract<br><br>p.2 abstract
**Introduction**					
Background rationale	2	Explain the scientific background and rationale for the investigation being reported	p.3		
Objectives	3	State specific objectives, including any prespecified hypotheses	p.3		
**Methods**					
Study Design	4	Present key elements of study design early in the paper	p.4		
Setting	5	Describe the setting, locations, and relevant dates, including periods of recruitment, exposure, follow-up, and data collection	p.4		

For peer review only

Participants	6	*(a) Cohort study* - Give the eligibility criteria, and the sources and methods of selection of participants. Describe methods of follow-up<br>*Case-control study* - Give the eligibility criteria, and the sources and methods of case ascertainment and control selection. Give the rationale for the choice of cases and controls<br>*Cross-sectional study* - Give the eligibility criteria, and the sources and methods of selection of participants<br><br>*(b) Cohort study* - For matched studies, give matching criteria and number of exposed and unexposed<br>*Case-control study* - For matched studies, give matching criteria and the number of controls per case		RECORD 6.1: The methods of study population selection (such as codes or algorithms used to identify subjects) should be listed in detail. If this is not possible, an explanation should be provided.<br><br>RECORD 6.2: Any validation studies of the codes or algorithms used to select the population should be referenced. If validation was conducted for this study and not published elsewhere, detailed methods and results should be provided.<br><br>RECORD 6.3: If the study involved linkage of databases, consider use of a flow diagram or other graphical display to demonstrate the data linkage process, including the number of individuals with linked data at each stage.	6.1 p.5<br><br>**6.2 p.5**<br>Toelichting.<br><br>6.3. figure 2, p.21
Variables	7	Clearly define all outcomes, exposures, predictors, potential confounders, and effect modifiers. Give diagnostic criteria, if applicable.	p.7-8	RECORD 7.1: A complete list of codes and algorithms used to classify exposures, outcomes, confounders, and effect modifiers should be provided. If these cannot be reported, an explanation should be provided.	p.7-8
Data sources/ measurement	8	For each variable of interest, give sources of data and details of methods of assessment (measurement).<br>Describe comparability of assessment methods if there is more than one group	p.7-8		

For peer review only

Bias	9	Describe any efforts to address potential sources of bias	p. 6		
Study size	10	Explain how the study size was arrived at	p. 4, 6		
Quantitative variables	11	Explain how quantitative variables were handled in the analyses. If applicable, describe which groupings were chosen, and why	p. 7		
Statistical methods	12	(a) Describe all statistical methods, including those used to control for confounding<br>(b) Describe any methods used to examine subgroups and interactions<br>(c) Explain how missing data were addressed<br>(d) *Cohort study* - If applicable, explain how loss to follow-up was addressed<br>*Case-control study* - If applicable, explain how matching of cases and controls was addressed<br>*Cross-sectional study* - If applicable, describe analytical methods taking account of sampling strategy<br>(e) Describe any sensitivity analyses	p. 7		
Data access and cleaning methods		..		RECORD 12.1: Authors should describe the extent to which the investigators had access to the database population used to create the study population.	p. 6

For peer review only

				RECORD 12.2: Authors should provide information on the data cleaning methods used in the study.	
Linkage		..		RECORD 12.3: State whether the study included person-level, institutional-level, or other data linkage across two or more databases. The methods of linkage and methods of linkage quality evaluation should be provided.	p. 6
**Results**					
Participants	13	(a) Report the numbers of individuals at each stage of the study (*e.g.*, numbers potentially eligible, examined for eligibility, confirmed eligible, included in the study, completing follow-up, and analysed)<br>(b) Give reasons for non-participation at each stage.<br>(c) Consider use of a flow diagram	p.9	RECORD 13.1: Describe in detail the selection of the persons included in the study (*i.e.,* study population selection) including filtering based on data quality, data availability and linkage. The selection of included persons can be described in the text and/or by means of the study flow diagram.	p. 6 and figure 2. P21
Descriptive data	14	(a) Give characteristics of study participants (*e.g.*, demographic, clinical, social) and information on exposures and potential confounders<br>(b) Indicate the number of participants with missing data for each variable of interest<br>(c) *Cohort study* - summarise follow-up time (*e.g.*, average and total amount)	Table 1<br><br>p. 9		
Outcome data	15	*Cohort study* - Report numbers of outcome events or summary measures over time<br>*Case-control study* - Report numbers in each exposure	p. 9 and Table 1		

For peer review only

		category, or summary measures of exposure<br>*Cross-sectional study* - Report numbers of outcome events or summary measures			
Main results	16	(a) Give unadjusted estimates and, if applicable, confounder-adjusted estimates and their precision (e.g., 95% confidence interval). Make clear which confounders were adjusted for and why they were included<br>(b) Report category boundaries when continuous variables were categorized<br>(c) If relevant, consider translating estimates of relative risk into absolute risk for a meaningful time period	Table 2		
Other analyses	17	Report other analyses done—e.g., analyses of subgroups and interactions, and sensitivity analyses	Table 3		
**Discussion**					
Key results	18	Summarise key results with reference to study objectives	p.11		
Limitations	19	Discuss limitations of the study, taking into account sources of potential bias or imprecision. Discuss both direction and magnitude of any potential bias	p.11-12	RECORD 19.1: Discuss the implications of using data that were not created or collected to answer the specific research question(s). Include discussion of misclassification bias, unmeasured confounding, missing data, and changing eligibility over time, as they pertain to the study being reported.	p.11-12
Interpretation	20	Give a cautious overall interpretation of results considering objectives,	p.12-13		

For peer review only

		limitations, multiplicity of analyses, results from similar studies, and other relevant evidence			
Generalisability	21	Discuss the generalisability (external validity) of the study results	p.12-13		
**Other Information**					
Funding	22	Give the source of funding and the role of the funders for the present study and, if applicable, for the original study on which the present article is based	Information provided to journal		
Accessibility of protocol, raw data, and programming code		..	Information provided to journal	RECORD 22.1: Authors should provide information on how to access any supplemental information such as the study protocol, raw data, or programming code.	Information provided to journal

*Reference: Benchimol EI, Smeeth L, Guttmann A, Harron K, Moher D, Petersen I, Sørensen HT, von Elm E, Langan SM, the RECORD Working Committee. The REporting of studies Conducted using Observational Routinely-collected health Data (RECORD) Statement. *PLoS Medicine* 2015; in press.

*Checklist is protected under Creative Commons Attribution (CC BY) license.

For peer review only - http://bmjopen.bmj.com/site/about/guidelines.xhtml
